# Preparation of *E. coli* RNA polymerase transcription elongation complexes by selective photoelution from magnetic beads

**DOI:** 10.1016/j.jbc.2021.100812

**Published:** 2021-05-21

**Authors:** Eric J. Strobel

**Affiliations:** Department of Biological Sciences, The University at Buffalo, Buffalo, New York, USA

**Keywords:** transcription, RNA polymerase, DNA, RNA, transcription elongation complex, biotin, photocleavable biotin, selective photoelution, dU, deoxyuridine, NaOAc, sodium acetate, NEB, New England Biolabs, PC, photocleavable, RNAP, RNA polymerase, TBE, tris-borate-EDTA, TEC, transcription elongation complex, TEG, triethylene glycol

## Abstract

*In vitro* studies of transcription frequently require the preparation of defined elongation complexes. Defined transcription elongation complexes (TECs) are typically prepared by constructing an artificial transcription bubble from synthetic oligonucleotides and RNA polymerase. This approach is optimal for diverse applications but is sensitive to nucleic acid length and sequence and is not compatible with systems where promoter-directed initiation or extensive transcription elongation is crucial. To complement scaffold-directed approaches for TEC assembly, I have developed a method for preparing promoter-initiated *Escherichia coli* TECs using a purification strategy called selective photoelution. This approach combines TEC-dependent sequestration of a biotin–triethylene glycol transcription stall site with photoreversible DNA immobilization to enrich TECs from an *in vitro* transcription reaction. I show that selective photoelution can be used to purify TECs that contain a 273-bp DNA template and 194-nt structured RNA. Selective photoelution is a straightforward and robust procedure that, in the systems assessed here, generates precisely positioned TECs with >95% purity and >30% yield. TECs prepared by selective photoelution can contain complex nucleic acid sequences and will therefore likely be useful for investigating RNA structure and function in the context of RNA polymerases.

The preparation of defined transcription elongation complexes (TECs) is important for *in vitro* biochemical, biophysical, and structural studies of RNA polymerases (RNAPs) and nascent RNA (1, 2, 3, 4, 5, 6, 7, 8, 9, 10, 11, 12, 13, 14, 15, 16, 17). The predominant approach for assembling defined TECs is to construct ternary complexes by sequentially adding nucleic acid and protein components to a reaction mixture (18, 19, 20). This approach for assembling TEC ‘scaffolds’ affords complete control over the identity of the complexes, which can be constructed with unnatural elements including nucleic acid modifications or a mismatched transcription bubble (20). For many applications, this approach is optimal; however, some biologically important transcription complexes may require cotranscriptional or promoter-directed assembly (21, 22, 23). In such cases, a method for purifying TECs after promoter-directed initiation and uninterrupted transcription elongation would be advantageous.

Although it is possible to ‘walk’ RNAPs to a defined DNA position by sequentially supplying NTP mixtures that omit one nucleotide at a time (20), this approach is not compatible with continuous transcription elongation and was not designed for the purpose of isolating pure TECs. Purifying defined, promoter-initiated TECs is difficult for two reasons: First, transcription initiation is not 100% efficient (24, 25, 26). Consequently, in a typical single-round *in vitro* transcription reaction, a substantial fraction of open promoter complexes will not convert to productive elongation complexes and some DNA may not contain an RNAP at all. Second, when an RNAP escapes a promoter, the unoccupied promoter can become a substrate for the formation of a new open complex depending on the conditions used for single-round transcription. If unaccounted for, this can result in a population of complexes that contains both a TEC and an open promoter complex. Here, I have addressed both of these challenges to develop a straightforward procedure for purifying precisely positioned *Escherichia coli* RNAP TECs after promoter-directed transcription initiation.

In this work, I describe the development and validation of a selective photoelution strategy for purifying promoter-initiated *E*. *coli* TECs. This approach is based on my previous observation that stalling *E*. *coli* RNAP at an internal desthiobiotin–triethylene glycol (TEG) (or biotin–TEG, as described here) lesion blocks binding to streptavidin-coated magnetic beads (27). I first show that TEC-dependent streptavidin bead exclusion can be coupled with reversible DNA immobilization using a 5’ photocleavable (PC) biotin so that TECs can be separated from both DNA without a TEC and excess transcription reaction components. Second, I show that the homogeneity of TEC preparations can be optimized using a DNA competitor strategy that prevents the formation of new open promoter complexes after transcription initiation. Last, I demonstrate the utility of selective photoelution by purifying TECs that contain a 273-bp DNA template and 194-nt structured RNA. Selective photoelution enriches TECs to >95% purity with >30% yield and can be performed in <4 h. Overall, this work establishes a straightforward approach for purifying promoter-initiated *E*. *coli* RNAP TECs that may be generalizable to other processive enzymes.

## Results

### Overview of the strategy for TEC purification by selective photoelution

**Figure 1:**
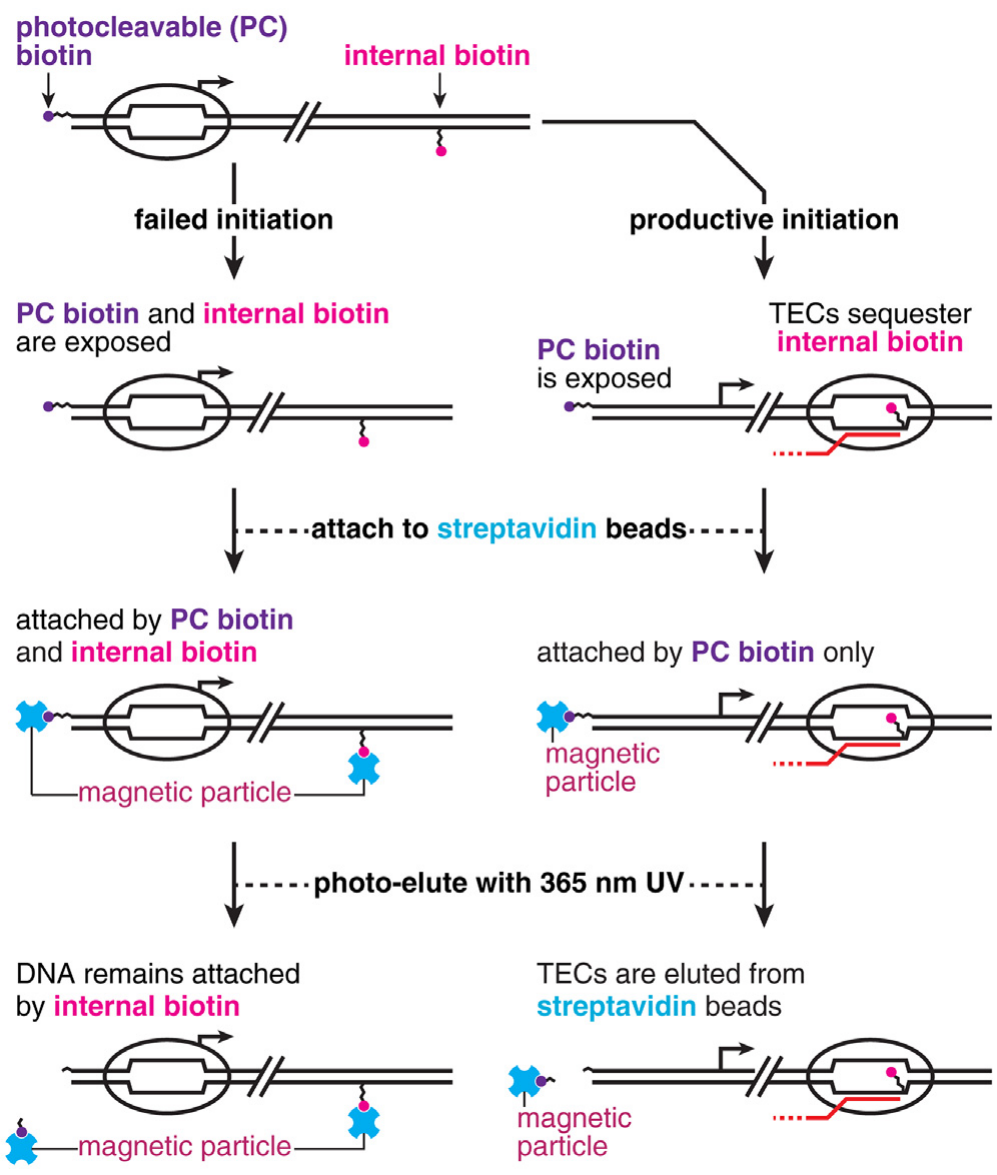
**Overview of the selective photoelution strategy for TEC purification.** The DNA template used for TEC purification contains two biotin modifications: an internal biotin–TEG modification in the transcribed DNA strand, which functions as an *Escherichia coli* RNAP stall site, and a 5’ PC biotin. If RNAP fails to escape the promoter, DNA is attached to streptavidin beads by both internal biotin–TEG and PC biotin and therefore remains attached to the beads after irradiation with 365-nm UV light. When RNAP escapes the promoter, the TEC stalls at and sequesters an internal biotin–TEG site so that the DNA is only attached to streptavidin beads by PC biotin. TECs can therefore be selectively eluted by 365-nm UV light. PC, photocleavable; RNAP, RNA polymerase; TEC, transcription elongation complex; TEG, triethylene glycol.

Purifying promoter-initiated TECs requires a fractionation approach that can separate DNA that contains TECs from nonproductive promoter-bound complexes and naked DNA. I previously observed that positioning *E*. *coli* RNAP at an internal desthiobiotin–TEG stall site blocks attachment to streptavidin-coated magnetic beads (27). TEC-dependent streptavidin bead exclusion provides a basis for TEC purification but must be coupled with a gentle buffer exchange to deplete excess transcription reaction components. To this end, I designed the following purification strategy (Fig. 1): A DNA template containing both an internal biotin–TEG transcription stall site and a 5’ PC biotin modification (28) is *in vitro*–transcribed under single-round conditions, and the reaction is mixed with streptavidin-coated magnetic beads. The 5’ PC biotin modification is unconditionally exposed so that all DNA can be immobilized and washed. In contrast, the biotin–TEG modification is conditionally exposed because stalled TECs block streptavidin binding. If biotin–TEG is not sequestered by a TEC, the DNA is attached to a magnetic bead by both biotin–TEG and 5’ PC biotin. However, when a stalled TEC sequesters the biotin–TEG modification, the DNA is immobilized by 5’ PC biotin alone. Consequently, DNA that contains a TEC can be selectively eluted by 365-nm UV light, and DNA without a TEC is retained in the bead pellet by biotin–TEG. The sections below describe the development and optimization of this procedure.

### TECs can be efficiently enriched by streptavidin bead exclusion

**Figure 2:**
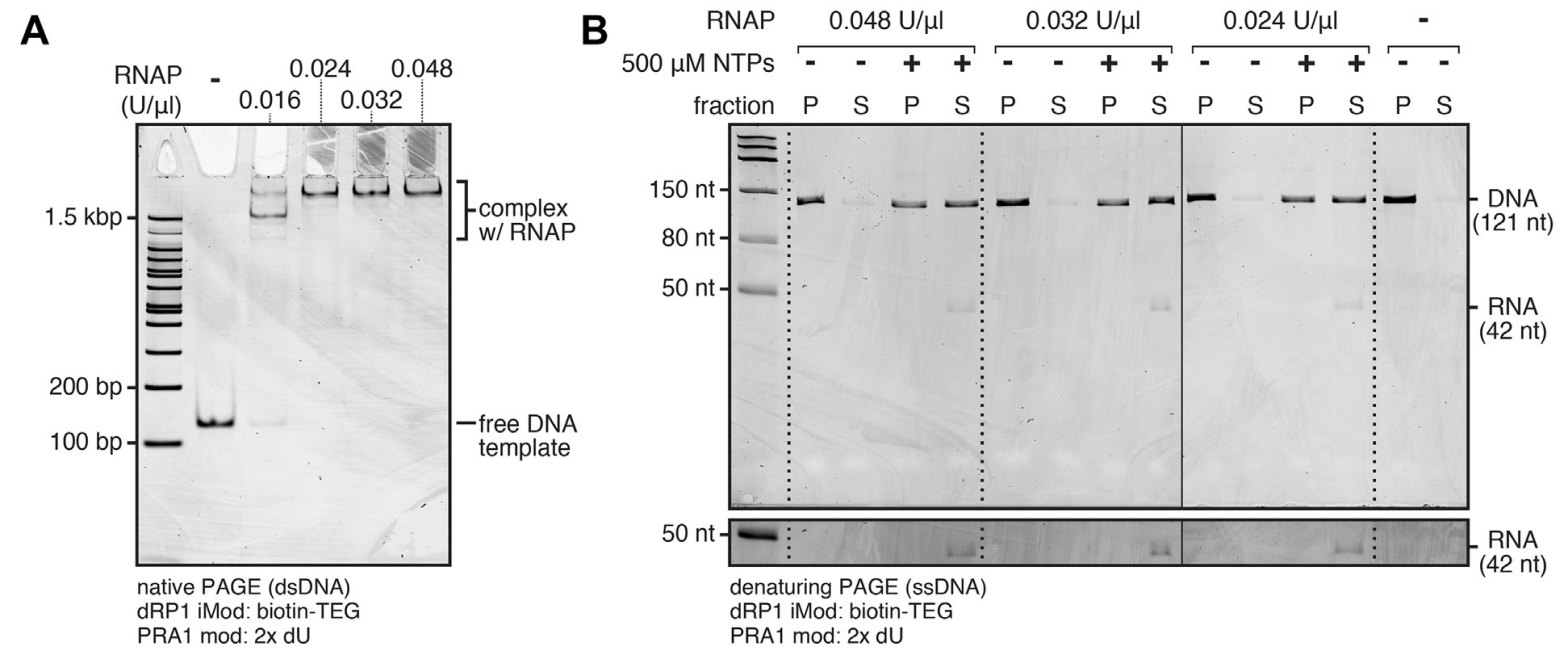
**Transcription-dependent exclusion of internal biotin–TEG–modified DNA from streptavidin-coated magnetic beads.***A*, EMSA of open complexes formed with 5 nM DNA template and variable concentrations of *Escherichia coli* RNAP. *B*, denaturing PAGE of an internal biotin–TEG–modified DNA template that was fractionated using streptavidin-coated magnetic beads after transcription with variable concentrations of *E*. *coli* RNAP with and without NTPs. The *solid vertical line* between the 0.032 and 0.024 U/μl samples indicates a gel splice. The grayscale on the lower cut-out is adjusted to better show the RNA band; the full gel is shown with this darker grayscale setting in Fig. S6*A*. The experiment in panel *A* was performed once to set conditions for panel *B*. The gels shown in panel *B* are representative of two independent replicates. P, pellet; RNAP, RNA polymerase; S, supernatant; TEG, triethylene glycol.

The purification strategy shown in Figure 1 depends on the efficient separation of DNA that does not contain a TEC from DNA with a TEC by exclusion of the latter from streptavidin-coated magnetic beads. To validate streptavidin bead exclusion as an approach for purifying TECs, I assessed the efficiency at which DNA containing a biotin–TEG transcription stall site was attached to streptavidin beads without and with single-round transcription. The P_RA1_ promoter used for this and all subsequent assays is a derivative of λP_R_ (positions −1 to −35, A-29C) that contains the proximal UP element subsite (29) of the T7A1 promoter (positions −36 to −50) and deoxyuridine (dU) nucleotides at −13 and −30, which are used in a footprinting assay below. λP_R_ was chosen as the primary basis of P_RA1_ because of its long open complex lifetime (30, 31). Without transcription, ∼97% of DNA was immobilized regardless of RNAP concentration (Fig. 2, *A* and *B*). This indicates that nonspecific binding by excess RNAP does not interfere with DNA immobilization. The ∼3% of DNA that does not bind streptavidin beads is likely a population in which the biotin–TEG modification was either inactivated or not incorporated during oligonucleotide synthesis. When single-round transcription was initiated, ∼55% of DNA was excluded from the streptavidin beads in all conditions, and the expected 42-nt transcript was observed only in the supernatant (Fig. 2*B*). These data show that streptavidin bead exclusion can be used to efficiently isolate TECs.

### TECs can be selectively eluted from streptavidin-coated magnetic beads

**Figure 3:**
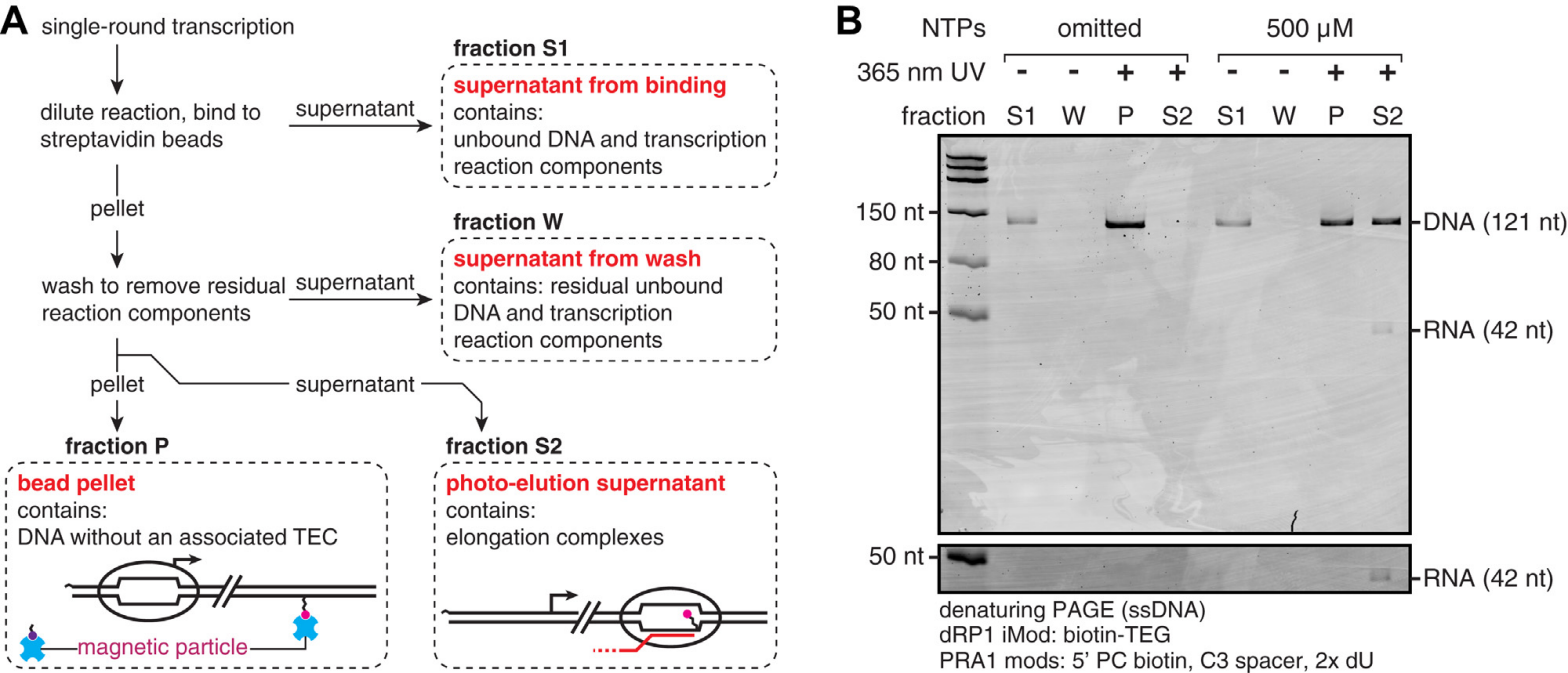
**Selective elution of roadblocked TECs from streptavidin beads.***A*, workflow of the TEC purification scheme. The source of each fraction (S1, W, P, S2) taken for the experiment in panel *B* is indicated. *B*, denaturing PAGE analysis of the TEC purification procedure shown in panel *A*. The grayscale on the lower cut-out is adjusted to better show the RNA band; the full gel is shown with this darker grayscale setting in Fig. S6*B*. The gel shown in panel *B* is representative of two independent replicates. TECs, transcription elongation complexes.

After confirming that TECs can be enriched by streptavidin bead exclusion, I evaluated the feasibility of the selective photoelution strategy shown in Figure 1. This proof-of-principle experiment used 5 nM DNA template and 0.024 U/μl RNAP in a 25 μl reaction to saturate transcription (Fig. 2*A*); further optimizations are described in the next section of the Results. TEC purification was performed as follows, and supernatant fractions were collected at key steps to monitor the efficiency of the procedure (Fig. 3*A*, see Experimental procedures for complete details): Open promoter complexes were formed without or with NTPs, and single-round transcription was initiated by adding MgCl_2_ and rifampicin. After 2 min of transcription, the reaction was diluted 9-fold, mixed with 25 μl of 1 μg/μl streptavidin beads, and incubated for 1 h at room temperature (RT) with rotation. The supernatant from this bead binding step was collected as fraction S1 (Fig. 3*A*). The beads were washed with 250 μl of transcription buffer supplemented with 1 mM MgCl_2_, and the wash supernatant was collected as fraction W (Fig. 3*A*). The beads were then resuspended in 25 μl of the same wash buffer and irradiated with 365-nm UV LEDs (∼10 mW/cm^2^ from four directions, Fig. S1) for 5 min. The bead pellet was collected as fraction P and the supernatant as fraction S2 (Fig. 3*A*). In both the absence and presence of NTPs, ∼17% of DNA was lost in fraction S1, presumably because the binding reaction was diluted to avoid crosslinking the beads with doubly biotinylated DNA (Fig. 3*B*). No nucleic acids were detected in fraction W (Fig. 3*B*). In the absence of NTPs, ∼2% of bead-bound DNA was eluted into fraction S2 upon 365-nm UV irradiation (Fig. 3*B*). This indicates that without transcription, DNA is retained on the beads by the internal biotin–TEG modification. When NTPs were included to permit transcription, ∼46% of bead-bound DNA was eluted into fraction S2 along with the expected 42-nt RNA transcript (Fig. 3*B*). This analysis validates selective photoelution as a TEC purification strategy.

### Scavenging free RNAP holoenzyme improves TEC homogeneity

**Figure 4:**
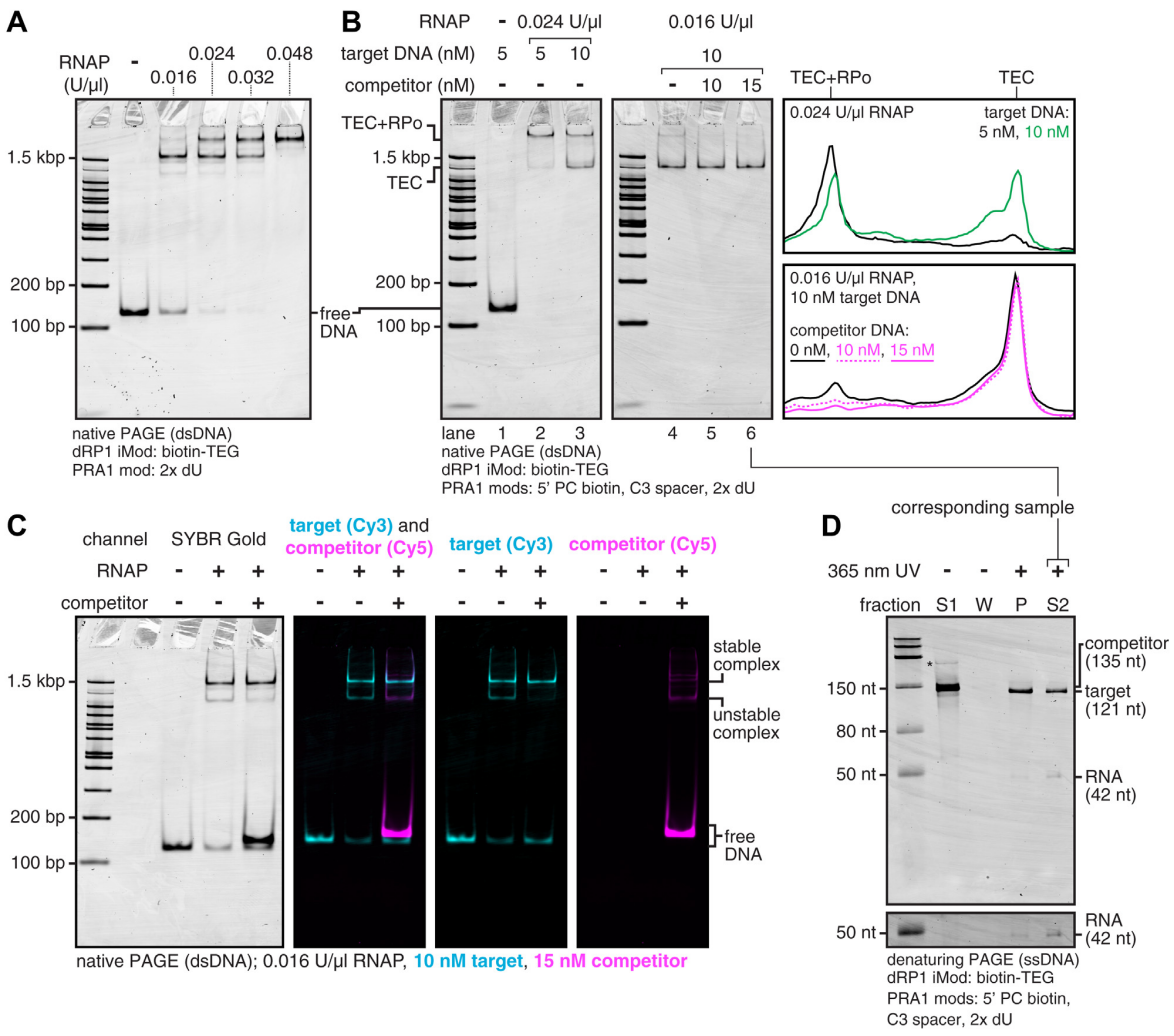
**Optimization of TEC purification.***A*, EMSA of open complexes formed with 10 nM DNA template and variable concentrations of *Escherichia coli* RNAP. *B*, EMSA of TECs purified with variable DNA template, RNAP, and competitor DNA template concentrations. The assay shown in Figure 5 revealed the slow-migrating band to be TECs with an associated open promoter complex (RPo), and the fast-migrating band to be pure TECs. Independent TEC preparations using the conditions from lanes 4, 5, and 6 are shown in Fig. S2. Note that when preparing TECs with 10 nM DNA, the amount of streptavidin beads was doubled, and the binding reaction diluted to 500 μl accordingly (Experimental procedures). *C*, EMSA of sequentially formed open complexes with Cy3-labeled target and Cy5-labeled competitor DNA. *D*, denaturing PAGE of purification fractions for TECs prepared using the conditions in lane 6 of panel *B*. Fractions S1, W, P, and S2 were taken as indicated in Figure 3*A*. The *asterisk* indicates a minor PCR product that was present in the competitor DNA template preparation. The grayscale on the lower cut-out is adjusted to better show the RNA band; the full gel is shown with this darker grayscale setting in Fig. S6*C*. The experiment in panel *A* was performed once to set conditions for panels *B*–*D*. The gels shown in panels *B*–*D* are representative of two independent replicates. RNAP, RNA polymerase; TECs, transcription elongation complexes.

The proof-of-principle TEC purification shown in Figure 3 used a saturating RNAP concentration to assess the efficiency limit of the preparation. However, limiting transcription to a single round with rifampicin inhibits new initiation but does not prevent new open promoter complexes (as opposed to heparin, which prevents new open complexes but could interfere with downstream applications (32)). These conditions are not optimal for preparing pure TECs because, after promoter escape, excess RNAP can bind unoccupied promoters to yield DNA that contains a TEC and an open complex. As expected, TECs that were prepared using excess RNAP migrated as two bands when assessed by an EMSA (Fig. 4*B*, lanes 2 and 3). Decreasing the RNAP:DNA ratio caused the fast-migrating band to become more prominent (Fig. 4, *A* and *B*, compare lanes 2, 3, and 4). The lowest RNAP:DNA ratio tested (0.016 U/μl RNAP with 10 nM DNA) yielded fast-migrating TECs with ∼94% purity (Fig. 4*B*, lane 4). The dependence of TEC homogeneity on the RNAP:DNA ratio suggests that fast-migrating complexes correspond to pure TECs and slow-migrating complexes correspond to TECs with an associated open complex.

To ensure consistent TEC homogeneity, I implemented a DNA competitor strategy to scavenge free RNAP holoenzyme before initiating transcription. Below, ‘target DNA’ is the doubly biotinylated DNA substrate for TEC purification (DNA template 2 in Table S2) and ‘competitor DNA’ is the DNA template used to scavenge RNAP holoenzyme (DNA template 3 in Table S2). Open promoter complexes are first formed on target DNA using 10 nM DNA template and 0.016 U/μl RNAP. The reaction is then mixed with competitor DNA, which contains a P_RA1_ promoter to scavenge RNAP holoenzyme in open complexes and a 1,N6-etheno-2’-deoxyadenosine stall site to retain RNAP after initiation. Visualization of this procedure using Cy3-labeled target DNA and Cy5-labeled competitor DNA (DNA templates 4 and 5 in Table S2, respectively) revealed the presence of both competitor-resistant target DNA:RNAP complexes and unstable complexes with a distinct mobility that were titrated to competitor DNA (Fig. 4*C*). The presence of excess free competitor DNA after open complex formation suggests that most RNAP is bound to DNA after this procedure (Fig. 4*C*). As expected, scavenging free RNAP yielded TEC preparations with >97% fast-migrating TECs (Fig. 4*B*, lanes 5 and 6, and Fig. S2). Competitor DNA was efficiently removed from the TEC preparations in fraction S1 (Fig. 4*D*). It was not possible to quantify the small fraction of target DNA in S1 because target and competitor DNA differ in length by only 14 nt (Fig. 4*D*). Approximately 37% of bead-bound DNA was released into fraction S2 after 365-nm UV irradiation (Fig. 4*D*).

**Figure 5:**
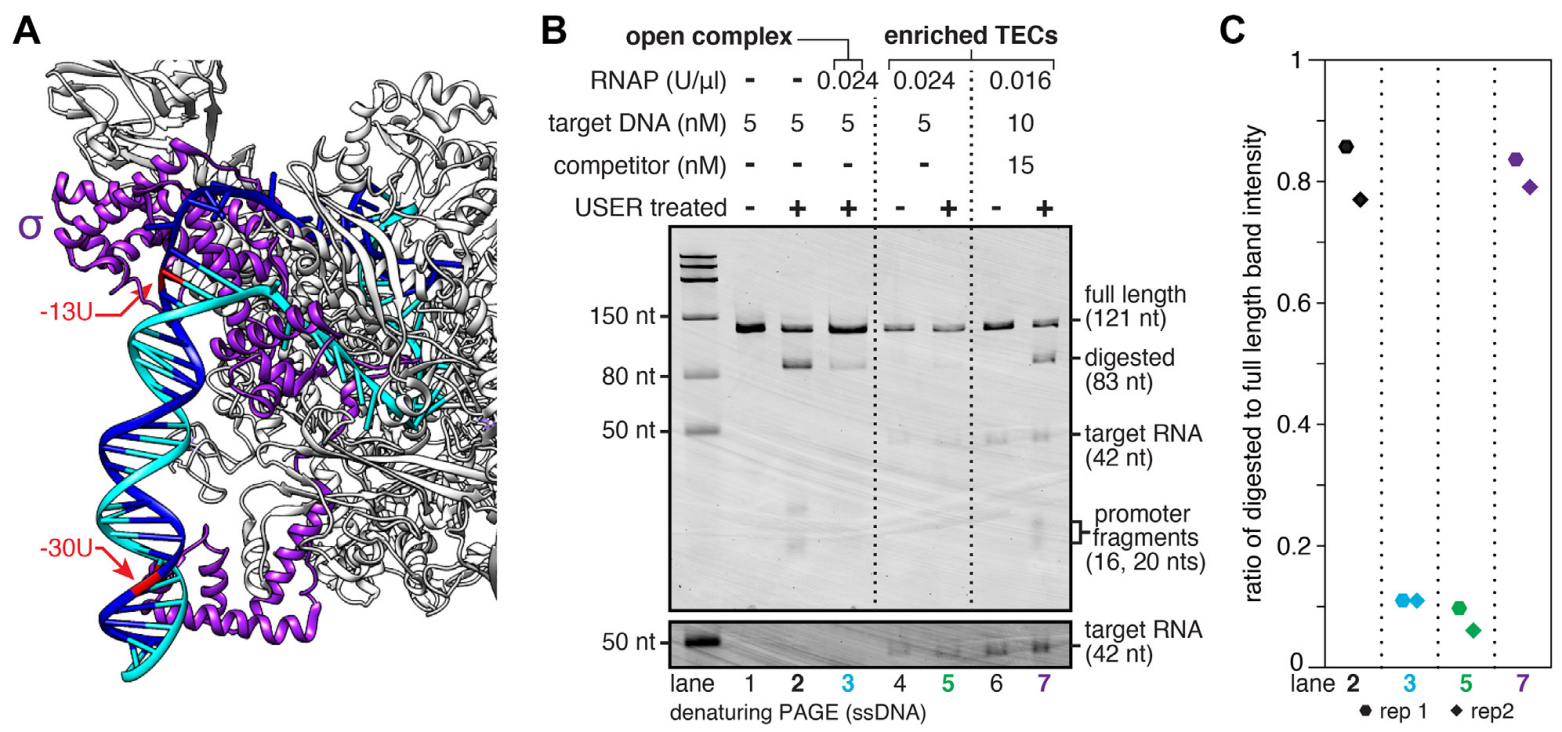
**USER footprinting to assess promoter accessibility.***A*, structure of *Thermus aquaticus* initiation complex (PDB: 4XLN) (5) annotated to indicate the position of P_RA1_ dU bases relative to direct contacts between σ and promoter DNA. *B*, USER footprinting assay to assess P_RA1_ promoter accessibility. Saturation of the P_RA1_ promoter with open complexes (conditions from Fig. 2*A*) attenuates USER digestion. TECs prepared under conditions that predominantly yield slow-migrating complexes (Fig. 4*B*, lane 2) inhibit USER digestion, indicating promoter occupation by open complexes. TECs prepared under conditions that yield >97% fast-migrating complexes (Fig. 4*B*, lane 6) are efficiently digested, indicating the absence of open complexes. The grayscale on the lower cut-out is adjusted to better show the RNA band; the full gel is shown with this darker grayscale setting in Fig. S6*D*. *C*, quantification of USER digestion for key lanes from panel *B* as the ratio of USER digested to full-length band intensity. The gel shown in panel *B* is representative of two independent replicates. TEC, transcription elongation complex.

To verify that fast- and slow-migrating complexes correspond to pure TECs and TECs with an associated open complex, respectively, I performed a ‘USER enzyme footprinting assay’ that directly assesses promoter accessibility. P_RA1_ open promoter complexes inhibit USER digestion because the dA-dU base pairs at −13 and −30 contact σ^70^ (Fig. 5, compare lanes 2 and 3 in Fig. 5, *B* and *C*). TECs that were prepared using conditions that yield slow-migrating complexes severely attenuated USER digestion (Fig. 5, *B* and *C*; lane 5). In contrast, TECs prepared using conditions that yield >97% fast-migrating complexes were digested as effectively as naked DNA (Fig. 5, *B* and *C*; lane 7). This supports the interpretation that the fast-migrating band in TEC preparations corresponds to pure TECs that do not contain an associated open complex. Taken together, these data establish a method for isolating high-purity TECs by selective elution from magnetic beads.

### Preparation of TECs that contain a 194-nt structured RNA

**Figure 6:**
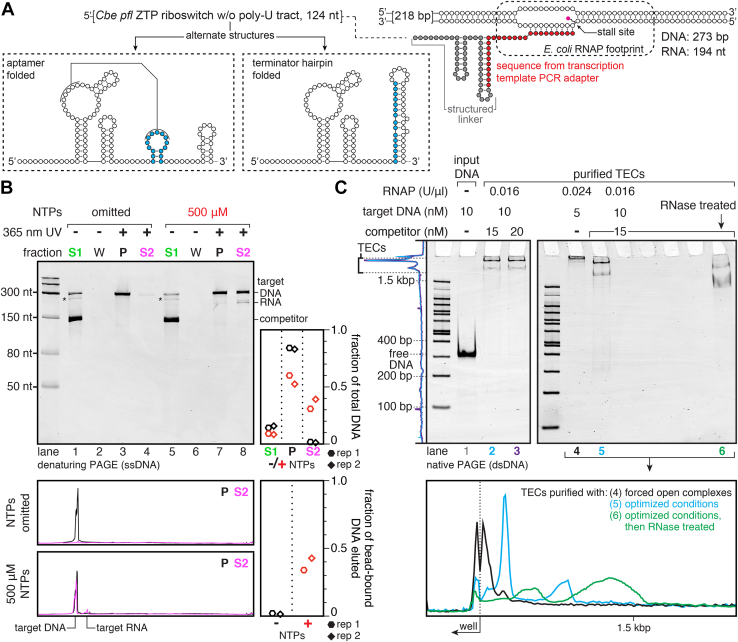
**Purification of TECs containing a 273-bp DNA template and 194-nt RNA.***A*, overview of the expected TECs. The target RNA contains a variant of the *Clostridium beijerinckii pfl* ZTP riboswitch with the poly-U tract removed to inactivate the transcription terminator. The *pfl* riboswitch is tethered to the TEC using a structured linker that sequesters a segment of the transcript derived from a PCR adapter sequence. *B*, denaturing PAGE analysis of fractions taken during purification of the TECs shown in panel *A*. Fractions S1, W, P, and S2 were taken as indicated in Figure 3*A*. The *asterisks* indicate a minor PCR product that was present in the competitor DNA template preparation. Intensity traces of the target DNA and target RNA bands in fractions P and S2 for each NTP condition are shown below the gel. The plots show the distribution of target DNA across fractions S1, P, and S2, and the fraction of bead-bound DNA that was eluted into fraction S2 after irradiation with 365-nm UV light. *C*, EMSA analysis of purified TECs. TECs purified using the validated reaction conditions, or with additional competitor DNA, migrate as two major bands (lanes 2, 3, and 5), which may correspond to the alternate riboswitch folds shown in panel *A*. TECs in lane 4 were prepared using conditions that favor the formation of new open complexes after promoter escape. TECs in lane 6 were prepared using the optimized conditions and treated with a mixture of RNase I, RNaseA, and RNase T1. Empty wells were left between lanes 5 and 6 to avoid RNase cross-contamination. The intensity traces to the *left* of the gel are for lanes 2 and 3. The intensity traces below the gels are for lanes 4, 5, and 6; for accurate comparison of mobility, these intensity traces were aligned using the sample well (see Experimental procedures). The gels shown in panels *B* and *C* are representative of two independent replicates. The replicate of lanes 4, 5, and 6 in panel *C* is shown in Fig. S4*A* to illustrate the reproducibility of the complex mobilities. Panels *B* and *C* (lanes 1, 2, and 3) are also shown in Fig. S4, *C* and *D* with the grayscale adjusted to show trace impurities in the preparation.

The experiments above used a short DNA template encoding a 42-nt unstructured RNA to simplify method development. To show that selective photoelution is compatible with a more complex system, I purified TECs that contain a variant of the *Clostridium beijerinckii pfl* ZTP riboswitch (33) in which the transcription terminator was inactivated by omitting its poly-U tract (Fig. 6*A*, Fig. S3). The *pfl* riboswitch was tethered to TECs using a structured linker that sequesters RNA transcribed from a priming site used during DNA template preparation (Fig. 6*A*, Fig. S3). The selective photoelution procedure efficiently separated DNA without a TEC from DNA with a TEC (Fig. 6*B*). Without transcription, <2% of bead-bound DNA was eluted into fraction S2. In contrast, transcription caused the release of 34% and 42% of bead-bound DNA into fraction S2 in a pair of replicates (Fig. 6*B*). This corresponds to a yield of 31% and 39% of the total input DNA (Fig. 6*B*). ZTP riboswitch TECs migrated as two distinct bands when assessed by EMSA (Fig. 6*C*, lanes 2, 3, 5, and Fig. S4*A*, lane 2). Three lines of evidence suggest that this heterogeneity is caused by the nascent transcript folding into alternate structures (34) and not by multiple RNAPs associating with a single DNA template: First, increasing the concentration of competitor DNA had no effect on complex mobility (Fig. 6*C*, compare lanes 2 and 3). Second, TECs that were prepared using a high RNAP:DNA ratio to force new open complexes to form after transcription initiation migrated extremely slowly as a single band that was distinct from both bands in the optimized preparation (Fig. 6*C*, compare lanes 4 and 5; Fig. S4*A*, compare lanes 1 and 2). Third, RNase treatment both shifted and changed the ratio of the slow- and fast-migrating ZTP riboswitch TECs (Fig. 6*C*, compare lanes 5 and 6; Fig. S4*A*, compare lanes 2 and 3). These data demonstrate that TEC purification by selective photoelution from streptavidin-coated magnetic beads is compatible with complex target sequences.

## Discussion

I have described an *in vitro* method for purifying promoter-initiated *E*. *coli* RNAP TECs using a new selective photoelution strategy. Selective photoelution leverages the ability of *E*. *coli* RNAP to block streptavidin binding when stalled at an internal biotin–TEG DNA modification as a means for TEC enrichment. By coupling TEC-dependent exclusion from streptavidin beads with reversible immobilization using a separate PC biotin moiety, high-purity TECs can be efficiently isolated from an *in vitro* transcription reaction. This approach is an alternative to established scaffold-based methods for purifying defined TECs that will be useful for constructing RNAP complexes that are sensitive to cotranscriptional processes such as RNA folding (35) or transcription pausing (22), or that require promoter-directed initiation (21, 23). Importantly, the TEC purification method described above can be performed with commercially available reagents and, with the exception of an inexpensive custom-built 365-nm UV irradiator (Fig. S1), common laboratory equipment.

The most common method for preparing defined *E*. *coli* RNAP TECs *in vitro* is a promoter-independent nucleic acid scaffold strategy in which single-stranded DNA, RNA, and RNAP are assembled into an artificial transcription bubble (20). Selective photoelution is fundamentally different from scaffold-based approaches for purifying TECs because RNAP undergoes promoter-directed initiation and elongates continuously to a defined DNA template position. Because selective photoelution isolates TECs that have been generated through natural pathways, its advantages complement scaffold-based approaches in two primary ways: First, scaffold-directed TEC assembly is typically performed with <70-nt DNA oligos and <20-nt RNA oligos (with several notable exceptions (7, 8, 11, 14, 15, 16)) because nucleic acid structure can interfere with complex assembly. In contrast, selective photoelution was optimized using TECs that contained a 121-bp DNA and 42-nt RNA (Fig. 4) and performed as efficiently when applied to TECs that contained a 273-bp DNA and a highly structured 194-nt riboswitch RNA (Fig. 6). Second, the TECs that are isolated by selective photoelution are generated by single-round *in vitro* transcription and will likely be useful for preparing complexes that contain cotranscriptionally folded RNA or require cotranscriptional assembly. An important limitation of the selective photoelution method is that the resulting roadblocked TECs are not transcription competent because of unnatural nucleic acid chemistry at the RNAP active center. In contrast, nucleic acid scaffolds enable TECs to be assembled with diverse configurations at the RNAP active center (3, 6). Selective photoelution will therefore be most useful for *in vitro* applications that require high-purity TECs containing complex nucleic acid sequences or structures but which are agnostic to the configuration of the RNAP active center.

The selective photoelution method described here consistently produces TECs with >95% purity and >30% yield. Two main impurities are present in these TEC preparations: First, even when TECs were purified using conditions that minimize free RNAP holoenzyme, ∼3% contained an associated open promoter complex (Fig. 4*B*). Given that the optimized conditions (10 nM target DNA, 0.016 U/μl RNAP, 15 nM competitor DNA) already contain a large excess of the competitor DNA template used to scavenge free RNAP holoenzyme (Fig. 4*C*), this appears to be an efficiency limit of the preparation that cannot be addressed with a reasonable amount of competitor DNA. Technically, these complexes do contain the desired TEC, and whether the presence of an open complex on the same DNA molecule constitutes an impurity will depend on the application at hand. Second, in the absence of TECs, ∼1.5% of bead-bound DNA was released into the supernatant upon 365-nm UV irradiation (Fig. 6*B*). This likely reflects a population of DNA template molecules that contain a functional 5’ PC biotin modification but in which the internal biotin–TEG modification was inactivated or was not incorporated during oligonucleotide synthesis. However, this measurement reflects the amount of free DNA eluted from beads in the absence of TECs and is therefore an upper bound for free DNA impurity. Given that the presence of TECs increased the amount of UV-eluted DNA to 34 to 42% of the fraction that had bound to the beads, these preparations contained a maximum of ∼5% free DNA. In practice, the amount of free DNA in a TEC preparation will be reduced by the efficiency of TEC formation (∼50% in this case) if the internal biotin–TEG was simply damaged during synthesis and can still function as a transcription stall site. Importantly, these impurities are minor and predictable and can be easily assessed using the same denaturing PAGE and EMSA approaches that were used to validate the method here.

Fundamentally, selective photoelution is a strategy for enriching macromolecular complexes based on their footprint. In principle, this approach should be applicable to other processive enzymes that block streptavidin binding when stably positioned at a biotin attachment point. Furthermore, the selective photoelution approach illustrates how footprint-based sequestration of functionalized DNA elements can enable sophisticated manipulations of macromolecular complexes.

## Experimental procedures

### Oligonucleotides

All oligonucleotides were purchased from Integrated DNA Technologies. A detailed description of all oligonucleotides including sequence, modifications, and purifications is presented in Table S1. Out of an abundance of caution, oligonucleotides and DNA preparations that contained a 5’ PC biotin modification were handled under low-intensity 592-nm light from SimpleColor Amber LEDs (Waveform Lighting) set to 40% intensity using a FilmGrade Flicker-Free LED Dimmer (Waveform Lighting) and stored as single-use aliquots. Cy3- and Cy5-labeled oligonucleotides and DNA preparations were handled under low-intensity room light and stored as single-use aliquots.

### Proteins

Q5U High-Fidelity DNA Polymerase, Q5 High-Fidelity DNA Polymerase, Vent (exo-) DNA polymerase, *Sulfolobus* DNA Polymerase IV, *E*. *coli* RNA Polymerase holoenzyme, Thermolabile Proteinase K, Thermolabile USER II Enzyme, Thermolabile Exonuclease I, and RNase I_f_ were purchased from New England Biolabs (NEB). RNase Cocktail was purchased from Thermo Fisher Scientific.

### DNA template preparation

Several DNA template preparation protocols were performed depending on the requirements of oligonucleotide modifications. For simplicity, each possible processing step is detailed below in general terms, and Table S2 provides details on the oligonucleotides and specific processing steps used for every DNA template preparation in this work. All DNA templates that required translesion synthesis were assessed by both denaturing and nondenaturing PAGE quality control analyses, which are shown in Figs. S4*B* and S5.

#### PCR amplification

DNA templates with dU bases were prepared using Q5U High-Fidelity DNA Polymerase (NEB) in reactions that contained 1X Q5U Buffer (NEB), 0.2 mM dNTPs (Invitrogen), 0.25 μM forward primer, 0.25 μM reverse primer, 20 pM template DNA, and 0.02 U/μl Q5U. Details of the oligonucleotides used for each DNA template preparation are available in Tables S1 and S2. DNA templates that did not contain dU bases were prepared in a reaction that used Q5 High-Fidelity DNA Polymerase (NEB) but was otherwise identical. Typically, six 100-μl reaction volumes were prepared on ice and aliquoted into 200-μl thin-walled tubes. The thermal cycling protocol was as follows: 98 °C for 30 s, [98 °C for 10 s, 65 °C for 20 s, 72 °C for 20 s] × 30 cycles, 72 °C for 5 min, and hold at 10 °C.

#### Translesion synthesis

After PCR amplification, DNA templates that required translesion synthesis were purified using a QIAquick PCR Purification Kit (Qiagen) (one column per 100-μl reaction) according to the manufacturer’s protocol and eluted in 50 μl of buffer EB (10 mM Tris, pH 8.5); for some DNA template preparations, one negative control reaction was eluted in 30 μl of buffer EB and not processed further. This PCR purification step is required to exchange the reaction buffer and deplete dNTPs. 500 μl translesion synthesis reactions contained 1X ThermoPol Buffer (NEB), 200 μM dGTP (Invitrogen), 200 μM dTTP (Invitrogen), 200 μM 2-Amino-dATP (TriLink BioTechnologies), 200 μM 5-propynyl-dCTP (TriLink BioTechnologies), 0.02 U/μl Vent (exo-) DNA Polymerase (NEB), and 0.02 U/μl *Sulfolobus* DNA Polymerase IV (NEB) and were split into 100-μl reaction volumes in 200-μl thin-walled tubes. Translesion synthesis reactions were incubated at 55 °C in a thermal cycler with a heated lid set to 105 °C for 1 h.

#### DNA purification by agarose gel extraction

Agarose gel purification was performed essentially as described previously (27). Five 100-μl PCR or translesion synthesis reactions were pooled and ethanol precipitated by adding 50 μl of 3M sodium acetate (NaOAc) (pH 5.5) and 1 ml of cold 100% ethanol and chilled at −70 °C for 30 min before centrifugation at 18,500*g* and 4 °C for 30 min. Pelleted DNA was washed with 1 ml of cold 70% ethanol, air-dried, and resuspended in 30 μl of 10 mM Tris HCl (pH 8.0). Samples were mixed with 6X XC-Only SDS DNA Loading Dye (30% glycerol, 10 mM Tris HCl (pH 8.0), 0.48% (w/v) SDS, 0.01% xylene cyanol FF) and run on a tris-borate-EDTA (TBE) 1% (wt/v) agarose gel that was prepared using SeaKem GTG Agarose (Lonza Bioscience) and contained 0.625 μg/ml ethidium bromide. All DNA was able to be excised without UV exposure because the comigration of ethidium bromide made the large quantity of DNA readily observable under visible spectrum light. DNA was purified using a QIAquick Gel Extraction Kit (Qiagen) according to the manufacturer’s protocol, except that agarose gel slices were melted at 30 °C and the DNA was eluted in 30 μl of 10 mM Tris HCl (pH 8.0). DNA concentration was quantified using a Qubit 4.0 Fluorometer (Invitrogen) with the Qubit dsDNA Broad Range Assay Kit (Invitrogen) according to the manufacturer’s protocol.

#### DNA cleanup by exonuclease digestion

To minimize light exposure, all DNA containing light-sensitive modifications was purified using an alternate protocol that used exonuclease digestion to degrade trace amounts of excess primers in place of gel extraction. This procedure was possible because all DNA was prepared using an oligonucleotide or previously gel-purified linear dsDNA as the PCR template, so it was not necessary to remove plasmid DNA. PCR or translesion synthesis reactions were pooled, and 0.5 μl of Thermolabile Exonuclease I (NEB) was added per 100-μl reaction volume. Reactions were incubated at 37 °C for 4 min using a thermal cycler and placed on ice. The thermal cycler block was set to 80 °C with a heated lid set to 105 °C and, after the block reached temperature, the reactions were returned to the block for 1 min to heat-inactivate the Thermolabile Exonuclease I. The reactions were then purified using a QIAquick PCR Purification Kit (up to 2.5 reactions per column) following the manufacturer’s protocol, except that two 750-μl Buffer PE washes were performed, and the samples were eluted in 50 μl of 10 mM Tris HCl (pH 8.0). The concentration of purified DNA was quantified using a Qubit 4.0 Fluorometer with the Qubit dsDNA Broad Range Assay Kit according to the manufacturer’s protocol. It is important to note that the Thermolabile Exonuclease I digestion was able to be performed immediately following translesion synthesis because primers were depleted to undetectable levels during the Q5U and Q5 PCRs (Fig. S5). My laboratory has observed that under PCR amplification conditions that do not lead to virtually complete primer depletion, the presence of excess oligonucleotides and dNTPs can lead to the formation of primer dimers because *Sulfolobus* DNA polymerase IV is active at 37 °C, which permits the formation of primer dimer products that do not form at higher temperatures. In DNA preparations where primers are not depleted during PCR, this issue is resolved simply by performing an additional PCR cleanup immediately after the translesion synthesis reaction to remove the DNA polymerases and free dNTPs.

### Sequences

DNA template sequences are available in Table S3. Fully annotated versions of the LZV3 test (https://benchling.com/s/seq-L8QDkyWpnMGkdqnkOXlO), competitor (https://benchling.com/s/seq-LI54uN63QQ9QyAAJXIAi), and ZTP riboswitch (https://benchling.com/s/seq-nGcTv06H6miX3D6hjpfp) DNA templates are available at Benchling.

### Open promoter complex formation assays

For Figures 2*A* and 4*A*, reactions containing 1X transcription buffer (20 mM Tris HCl (pH 8.0), 50 mM KCl, 1 mM DTT, 0.1 mM EDTA (pH 8.0)), 0.1 mg/ml BSA (Invitrogen), 5 nM (Fig. 2*A*) or 10 nM (Fig. 4*A*) DNA Template 1 (Table S2), and 0, 0.016, 0.024, 0.032, or 0.048 U/μl *E. coli* RNAP holoenzyme (NEB) were incubated in a dry bath set to 37 °C for 15 (Fig. 2*A*) or 20 (Fig. 4*A*) minutes to form open complexes. 15 μl of the 25-μl sample was mixed with 3 μl of 6X Native DNA Loading Dye (30% (v/v) glycerol, 10 mM Tris HCl (pH 8.0), 0.01% (w/v) bromophenol blue) and assessed by EMSA as described below in the section Analysis of transcription complexes by EMSA.

For Figure 4*C*, open complexes were prepared as described below in the section Purification of TECs by selective photoelution from streptavidin beads, except that NTPs were omitted from the reaction, DNA templates 4 and 5 (Table S2) were used as ‘target’ and ‘competitor’ DNA respectively, and the reactions were performed under low-intensity room light instead of 592-nm light. Reactions were staggered so that all sample handling reached completion simultaneously. 15 μl of the 25-μl sample was mixed with 3 μl of 6X Native DNA-Loading Dye and assessed by EMSA as described below in the section Analysis of transcription complexes by EMSA except that the gel was run in a dark room and nucleic acids were detected as follows: After the gel had run, it was transferred to a plastic dish containing 0.5X TBE, scanned sequentially on a Sapphire Biomolecular Imager (Azure Biosystems) using the 520 nm/565BP24 and 658 nm/710BP40 settings, removed from the imager, stained with 1X SYBR Gold in 0.5X TBE for 10 min and scanned again on a Sapphire Biomolecular Imager using the 488 nm/518BP22 setting.

### Preparation of streptavidin-coated magnetic beads

For TEC purifications, 2.5 μl or 5 μl of Dynabeads MyOne Streptavidin C1 beads per 25-μl sample volume was prepared in bulk: After placing the beads on a magnet stand and removing the storage buffer, the beads were resuspended in 500 μl of hydrolysis buffer (100 mM NaOH and 50 mM NaCl) and incubated at RT for 10 min with rotation. Hydrolysis buffer was removed, and the beads were resuspended in 1 ml of high salt wash buffer (50 mM Tris-HCl (pH 7.5), 2 M NaCl, 0.5% Triton X-100), transferred to a new tube, and washed by rotating for 5 min at RT. High salt wash buffer was removed, and the beads were resuspended in 1 ml of binding buffer (10 mM Tris HCl (pH 7.5), 300 mM NaCl, 0.1% Triton X-100), transferred to a new tube, and washed by rotating for 5 min at RT. After removing the binding buffer, the beads were washed twice with 500 μl of buffer T (1X transcription buffer supplemented with 0.1% Triton X-100) by resuspending the beads, transferring them to a new tube, washing with rotation for 5 min at RT, and removing the supernatant. After washing the second time with buffer T, the beads were resuspended to a concentration of ∼1 μg/μl in buffer T, split into 25- or 50-μl aliquots, and stored on ice until use.

For the internal biotin–TEG sequestration assay, Dynabeads MyOne Streptavidin C1 beads were prepared as described above, except that 10 μl of beads were used per 25 μl sample volume, and the beads were resuspended in 25 μl of buffer T per sample volume to ∼4 μg/μl and split into 25-μl aliquots.

### Internal biotin–TEG sequestration assay

For the internal biotin–TEG sequestration assay, all 25-μl transcription reactions contained 1X transcription buffer, 0.1 mg/ml BSA, and 5 nM DNA template 1 (Table S2). NTPs (GE Healthcare) were either omitted or included at 500 μM each; *E. coli* RNAP holoenzyme was either omitted or included at 0.024, 0.032, or 0.048 U/μl. At the time of preparation, the total reaction volume was 22.5 μl due to the omission of 10X start solution (100 mM MgCl_2_, 100 μg/ml rifampicin). For reach transcription reaction, 10 μl of 10 mg/ml Dynabeads MyOne Streptavidin C1 beads were prepared in advance as described above in the procedure Preparation of streptavidin-coated magnetic beads and stored on ice at a concentration of ∼4 μg/μl in 25 μl of buffer T until use.

Reactions were placed in a dry bath set to 37 °C for 15 min to form open promoter complexes; equilibrated streptavidin beads were moved from ice to RT at this time. 2.5 μl of 10X start solution was added to the transcription reaction, and transcription was allowed to proceed for 2 min during which an aliquot of beads was placed on a magnet stand to remove the buffer T used for bead storage. After 2 min of transcription, the pelleted beads were resuspended using the 25-μl transcription reaction, placed on a rotator, and incubated at RT for 30 min. The beads were then returned to the magnet stand, and the supernatant was separated from the pellet and added to 125 μl of stop solution (0.6 M Tris HCl (pH 8.0), 12 mM EDTA (pH 8.0)). To recover immobilized DNA, the bead pellet was resuspended in 25 μl of 95% formamide and 10 mM EDTA, heated at 100 °C for 5 min, placed on a magnet stand, and the supernatant was collected and added to 125 μl of stop solution.

Reactions were processed for denaturing Urea-PAGE by phenol:chloroform extraction and ethanol precipitation as follows: 150 μl of Tris (pH 8) buffered phenol:chloroform:isoamyl alcohol (25:24:1, v/v) (Thermo Scientific) was added to each sample. Samples were mixed by vortexing and inversion and centrifuged at 18,500*g* and 4 °C for 5 min. The aqueous phase was collected and transferred to a new tube. DNA was precipitated by adding 15-μl 3 M NaOAc (pH 5.5), 450-μl 100% ethanol, and 1.5-μl GlycoBlue Coprecipitant (Invitrogen) to each sample. The samples were chilled at −70 °C for 30 min and centrifuged at 18,500*g* and 4 °C for 30 min. After removing the ethanol, sample pellets were resuspended in 16 μl of formamide loading dye (90% (v/v) deionized formamide, 1X transcription buffer, 0.01% (w/v) bromophenol blue), heated at 95 °C for 5 min, and snap-cooled on ice for 2 min. Urea-PAGE was performed as described below in the section Denaturing Urea-PAGE.

### Purification of TECs by selective photoelution from streptavidin beads

Several protocols for purifying TECs were performed, both for the purpose of protocol development and for assessing the properties of TECs that were prepared in different ways. For simplicity, the final validated protocol is detailed first, and other variations that were performed are then described below with reference to the figure(s) in which each procedure was used. Preparations using the final protocol are shown in Figures 4, *B* and *D*, 5*B* and 6, *B* and *C*, Figs. S2, and S4. All sample handling was performed under low-intensity 592-nm amber light until the 365-nm UV irradiation step.

For TEC purification, ‘target DNA’ refers to DNA templates 2 or 6 (Table S2), which contain an internal biotin–TEG modification as the RNAP stall site and a P_RA1_ promoter with 5’ PC biotin and C3 Spacer modifications at the 5’ end and dU modifications at positions −13 and −30 of the promoter. ‘Competitor DNA’ refers to DNA template 3 (Table S2), which contains an unmodified P_RA1_ promoter and an internal 1,N6-etheno-2’-deoxyadenosine stall site. For each transcription reaction, 5 μl of 10 mg/ml Dynabeads MyOne Streptavidin C1 beads were prepared in advance as described above in the procedure Preparation of streptavidin-coated magnetic beads and stored on ice at a concentration of ∼1 μg/μl in 50 μl of buffer T until use. 25 μl *in vitro* transcription reactions containing 1X transcription buffer, 500 μM NTPs, 0.1 mg/ml BSA, 10 nM Target DNA, and 0.016 U/μl *E. coli* RNAP holoenzyme were prepared in a 1.7-ml microcentrifuge tube on ice; at this point, the total reaction volume was 20 μl due to the omission of competitor DNA and 10X start solution. Transcription reactions were placed in a dry bath set to 37 °C for 20 min to form open promoter complexes. After ∼17 min, a 1.7-ml microcentrifuge tube containing 2.5 μl of 150 nM competitor DNA was placed in the 37 °C dry bath to prewarm. After open complexes had formed on target DNA for 20 min, the 20-μl transcription reaction was transferred to the tube containing 2.5 μl of 150-nM competitor DNA; the final concentration of competitor DNA in the full 25-μl reaction volume was 15 nM. The transcription reaction was returned to 37 °C for an additional 20 min so that free RNAP holoenzyme formed open complexes with the competitor DNA. Buffer TR (1X transcription buffer supplemented with 10 μg/ml rifampicin and 0.1% Triton X-100) and streptavidin beads were removed from ice and kept at RT at this time. After ∼17 min, the streptavidin beads were pipetted to resuspend settled beads. After open complexes had formed on competitor DNA for 20 min, single-round transcription was initiated by adding 2.5 μl of freshly prepared 10X start solution. The sample was incubated at 37 °C for 2 min and gently diluted by adding 425 μl of RT buffer TR and mixing by pipetting. Buffer TR contains rifampicin to maintain single-round transcription conditions during bead binding. The purpose of diluting the reaction was to minimize the occurrence of bead cross-linking by DNA templates in which both biotin modifications were exposed; when bead binding was performed using a sample volume of 25 μl, substantial bead clumping was observed. No observable clumping occurred when the binding reaction was diluted 10- or 20-fold. The diluted 450-μl transcription reaction was then gently mixed with 50 μl of ∼1 μg/μl streptavidin beads by pipetting. The bead binding reaction was incubated in the dark at RT with rotation for 1 h. After 1 h, the bead binding mixture was spun briefly in a Labnet Prism mini centrifuge (Labnet International) by quickly flicking the switch on and off so that liquid was removed from the tube cap, but the speed of the mini centrifuge remained as low as possible. The 1.7-ml tube containing the bead binding reaction was placed on a magnet stand for at least 2 min to pellet the streptavidin beads on the tube wall, and the supernatant was carefully removed; this supernatant, which contains any reaction components that did not bind the beads including virtually all competitor DNA, is referred to as fraction S1. The 1.7-ml tube containing the beads was removed from the magnet stand, the beads were gently resuspended in 500 μl of buffer TM (1X Transcription Buffer supplemented with 1 mM MgCl_2_ and 0.1% Triton X-100) by pipetting, and the sample was returned to the magnet stand for 2 min to pellet the streptavidin beads before removing the supernatant; this supernatant, which contains residual reaction components, is referred to as fraction W. The streptavidin beads were then gently resuspended in 25 μl of buffer TM by pipetting so that the bead concentration was ∼2 μg/μl and placed in a custom-built 365-nm UV LED irradiator for 1.7-ml microcentrifuge tubes (Fig. S1, see Assembly and validation of a 365-nm UV microcentrifuge tube irradiator below for details) and exposed to ∼10 mW/cm^2^ 365-nm UV light from four directions for 5 min. After irradiation, the bead mixture was returned to the magnet stand for 1 min before collecting the supernatant; the pelleted beads, which contain DNA without a TEC and any TECs that were not eluted by 365-nm UV irradiation, are referred to as fraction P. The collected supernatant, which contains purified TECs, is referred to as fraction S2.

Several variations on this protocol were performed. In the proof-of-principle experiment in Figure 3*B*, the transcription reaction included 5 nM target DNA and 0.024 U/μl RNAP holoenzyme, competitor DNA was not included, half the amount of streptavidin beads (25 μg *versus* 50 μg) were used, and the volumes used for the bead binding and wash steps were halved (250 μl *versus* 500 μl). In Figures 4*B*, 5B, and Fig. S2*A*, TECs were prepared with variable RNAP, target DNA, and competitor DNA concentrations as annotated. The samples that used 5 nM target DNA were prepared with the protocol modifications described for the TEC preparation in Figure 3*B*. In Figure 6 and Fig. S4, TECs were prepared using the optimized protocol described above, a variation of the optimized protocol that used 20 nM competitor DNA, or conditions similar to those in Figure 3*B*, where 5 nM target DNA and 0.024 U/μl RNAP holoenzyme were used and competitor DNA was omitted, but bead binding was performed as described for the optimized protocol.

### Collection and processing of TEC purification fractions for denaturing PAGE

TEC purification fractions were prepared for denaturing PAGE as follows: Fractions S1 and W were mixed with 5 μl of 0.5 M EDTA (pH 8.0). To recover immobilized DNA in fraction P, the bead pellet was resuspended in 25 μl of 95% formamide and 10 mM EDTA, heated at 100 °C for 5 min, placed on a magnet stand, and the supernatant was collected and mixed with 125 μl of stop solution. Fraction S2 was mixed with 125 μl of stop solution. The volumes of fractions P and S2 were then raised to match the volume of fractions S1 and W (250 μl in Fig. 3*B* or 500 μl in Figs. 4*D* and 6*B*) by adding 1X transcription buffer. The fractions were extracted by adding an equal volume of Tris (pH 8) buffered phenol:chloroform:isoamyl alcohol (25:24:1, v/v), mixing by vortexing and inversion, and centrifuging at 18,500*g* and 4 °C for 5 min. The aqueous phase was collected and transferred to a new tube. When the volume of the extracted samples was 250 μl, nucleic acids were precipitated by adding 25-μl 3 M NaOAc (pH 5.5), 750 μl 100% ethanol, and 1.5 μl GlycoBlue Coprecipitant to each sample and chilling at −70 °C for 30 min. When the volume of the extracted samples was 500 μl, nucleic acids were precipitated by adding 50-μl 3 M NaOAc (pH 5.5), 350 μl 100% isopropanol, and 1.5 μl GlycoBlue Coprecipitant to each sample and chilling on ice for 30 min. The samples were centrifuged at 18,500*g* and 4 °C for 30 min, and the supernatant was removed. When isopropanol precipitations were performed, the pellet was washed once by adding 1 ml of cold 70% (v/v) ethanol, inverting the tube several times, centrifuging at 18,500*g* and 4 °C for 2 min, and removing the supernatant. After removing residual ethanol, the pellet was dissolved in 16 μl of formamide loading dye, heated at 95 °C for 5 min, and snap-cooled on ice for 2 min. Urea-PAGE was performed as described below in the section Denaturing Urea-PAGE.

### USER protection assay

For the USER protection assay, TECs were prepared in parallel using the final validated protocol (10 nM Target DNA (DNA template 2 in Table S2), 0.016 U/μl RNAP holoenzyme, and 15 nM competitor DNA (DNA template 3 in Table S2)) and conditions that mostly yielded a slow-migrating product when assessed by EMSA (5 nM target DNA, 0.024 U/μl RNAP holoenzyme, and no competitor DNA). One modification was made to the TEC purification protocol: Buffer TM was substituted with buffer TRM (1X Transcription Buffer, 1 mM MgCl_2_, and 10 μg/ml rifampicin) so that the TEC preparations contained 10 μg/ml rifampicin to prevent activity by any remaining open complexes during the USER digestion, which requires ATP and magnesium. During the bead-binding step of the TEC purification protocol, several control reactions were prepared. All 25-μl control reactions contained 1X transcription buffer, 0.1 mg/ml BSA, and 5 nM DNA template 2 (Table S2); the +RNAP control reaction contained 0.024 U/μl RNAP holoenzyme, which saturates the P_RA1_ promoter with open complexes (Fig. 2*A*). At the time of preparation, control reactions were 20 μl due to the omission of 10X rifampicin and 10X T4 DNA ligase buffer.

Upon elution from streptavidin beads, 25-μl aliquots of purified TECs were mixed with 2.5 μl of 10X T4 DNA ligase buffer and either placed directly in a dry bath set to 37 °C (−USER samples) or mixed with 0.5 μl of Thermolabile USER II Enzyme before being placed at 37 °C (+USER samples). In both cases, purified TECs were incubated at 37 °C for 30 min, which far exceeds the time required for complete USER digestion (<5 min). Control reactions were incubated at 37 °C for 15 min to form open complexes. Rifampicin was added to 10 μg/ml, and the reactions were incubated at 37 °C for five additional minutes before the addition of 2.5 μl of 10X T4 DNA ligase buffer and, in the case of the +USER samples, 0.5 μl of Thermolabile USER II Enzyme. The control reactions were incubated 37 °C for 30 min. USER digestion was stopped by the addition of 125 μl of stop solution, and the samples were processed for denaturing PAGE by phenol:chloroform extraction and ethanol precipitation as described in the section Internal biotin–TEG sequestration assay, resuspended in 16 μl of formamide loading dye, heated at 95 °C for 5 min, and snap-cooled on ice for 2 min. Urea-PAGE was performed as described below in the section Denaturing Urea-PAGE.

### TEC degradation assay

TECs were purified as described above; one reaction volume was removed from the *in vitro* transcription master mix before the addition of *E*. *coli* RNAP holoenzyme and kept on ice as a DNA-only control. Purified TECs were split into 25-μl aliquots and kept at RT. When included in the reaction, 1 μl of Thermolabile Proteinase K (NEB) was added and mixed with the sample immediately before the sample was placed at 37 °C. All samples were incubated at 37 °C for 30 min, and one sample was heat-inactivated by incubating at 65 °C in a thermal cycler with a heated lid set to 105 °C for 10 min. The time course was structured so that all sample handling reached completion simultaneously. 15 μl of each sample was gently mixed with 3-μl 6X native DNA loading dye and assessed by EMSA as described below in the section Analysis of transcription complexes by EMSA.

### RNA degradation assay

TECs were purified as described above and split into 25-μl aliquots from a common pool. RNA degradation was performed by adding 0.5 μl of RNase I_f_ (NEB) alone or in combination with 0.5 μl of RNase Cocktail (Thermo Fisher Scientific), which contains RNase A and RNase T1, and incubating at 37 °C for 15 min. The untreated control sample was kept at RT during this time. 15 μl of each sample was gently mixed with 3-μl 6X native DNA loading dye and assessed by EMSA as described below in the section Analysis of transcription complexes by EMSA.

### Analysis of transcription complexes by EMSA

Transcription complexes, including open promoter complexes and purified TECs, were assessed by EMSA as follows: For all samples, 15 μl of a 25-μl sample was mixed with 3 μl of 6X native DNA loading dye. Samples were loaded on a 0.5X TBE 5% polyacrylamide gel prepared for a Mini-PROTEAN Tetra Vertical Electrophoresis Cell (Bio-Rad) using ProtoGel acrylamide (National Diagnostics). Gels were immersed up to the wells in 0.5X TBE to minimize heating and run at RT at 45V for 1.5, 1.75, or 2 h. With the exception of experiments that used fluorophore-labeled DNA, EMSA gels were then stained with 1X SYBR Gold in 0.5X TBE for 10 min and scanned on a Sapphire Biomolecular imager using the 488 nm/518BP22 setting. Detection of fluorophore-labeled DNA is described above in the section Open promoter complex formation assays*.*

### Denaturing Urea-PAGE

Gels for Urea-PAGE were prepared at 10% or 12% polyacrylamide using the SequaGel UreaGel 19:1 Denaturing Gel System (National Diagnostics). The conditions used for denaturing PAGE were selected to ensure complete and consistent denaturation of dsDNA using a Mini-PROTEAN Tetra Vertical Electrophoresis Cell: The inner buffer chamber was filled completely with 1X TBE, but the outer buffer chamber contained enough 1X TBE to cover only ∼1 cm of the gel plates to reduce heat loss. Urea-PAGE gels were run at 480 V, which is 80% of the maximum voltage recommended for the Mini-PROTEAN system. Urea-PAGE gels were stained with 1X SYBR Gold (Invitrogen) in 1X TBE for 10 min and scanned on a Sapphire Biomolecular Imager using the 488-nm/518BP22 setting.

### Native PAGE for the analysis of purified DNA

Purified DNA was assessed using 1X TBE 8% polyacrylamide gels prepared for a Mini-PROTEAN Tetra Vertical Electrophoresis Cell (Bio-Rad) using ProtoGel (National Diagnostics) acrylamide and run at 100 V. Native PAGE gels were stained with 1X SYBR Gold in 1X TBE for 10 min and scanned on a Sapphire Biomolecular Imager using the 488-nm/518BP22 setting.

### Assembly and validation of a 365-nm UV microcentrifuge tube irradiator

To enable efficient 365-nm UV-induced cleavage of 5’ PC biotin in a magnetic bead mixture (which can reduce photocleavage efficiency due to light scattering), a custom 1.7-ml microcentrifuge tube irradiator was assembled using 365-nm real-UV LED Light Strips (Waveform Lighting, Product # 7021.65). The irradiator was designed such that each tube was irradiated by four individual segments of the LED light strip (three LEDs each, ∼10 mW/cm^2^ from ∼1 cm away from the tube) simultaneously. The irradiator was constructed using the 365-nm LED strips above, LED Strip to Strip Solderless Connectors (Waveform Lighting, Product # 3071), a Female DC Barrel Jack Plug Adapter (Waveform Lighting, Product # 7094), the tube-holder insert from a Beta Box for 1.5-ml microcentrifuge tubes (Fisher Scientific, Cat # 12-009-14), a Desktop AC Adapter (MEAN WELL, Cat # GST60A12-P1J), and a NEMA 5-15P to IEC320C13 Universal Power Cord (C2G, Product #03129). LED strips were mounted to the tube-holder insert using 12-mm VHB Double-Sided Foam Adhesive Tape 5952 (3M) and hardware including corner brackets, machine screws, washers, and T plates. A UVA/B light meter calibrated to 365 nm (General, Item # UV513AB) was used to assess 365-nm UV intensity.

To assess 365-nm UV-induced cleavage of 5’ PC biotin, 25-μl reactions containing 1X Transcription Buffer, 0.1 mg/ml BSA, and 5 nM primer PRA1_2dU_PCbio.F (Table S1) were mixed with streptavidin-coated magnetic beads (prepared as described above in Preparation of streptavidin-coated magnetic beads) to 2 μg/μl or 1 μg/μl and incubated at RT with rotation for 30 min. The supernatant from this binding reaction, which contains any oligonucleotide that did not bind the beads, was discarded, and the beads were resuspended in 25 μl of buffer T and exposed to 365-nm UV light at 10 mW/cm^2^ from four directions for 5 min. The samples were placed on a magnet stand, and the supernatant was collected in 125 μl of stop solution. To recover immobilized DNA, the bead pellet was resuspended in 25 μl of 95% formamide and 10 mM EDTA, heated at 100 °C for 5 min, and placed on a magnet stand, and the supernatant was collected and added to 125 μl of stop solution. Reactions were processed for denaturing Urea-PAGE by phenol:chloroform extraction and ethanol precipitation as described above in the section Internal biotin–TEG sequestration assay and resuspended in 7-μl formamide loading dye. Urea-PAGE was performed as described above in the section Denaturing Urea-PAGE.

### Quantification

Quantification of band intensity was performed using ImageJ 1.51s by plotting each lane, drawing a line at the base of each peak to subtract background, and determining the area of the closed peak. In Figure 2*B*, the fraction of DNA in the supernatant was calculated by dividing the supernatant DNA band intensity by the sum of the pellet and supernatant DNA band intensities. The fraction of DNA in the pellet is 1 minus the fraction of DNA in the supernatant. When quantifying TEC purification fractions in Figures 3*B*, 4*D*, and 6*B*, the fraction of bead-bound DNA that was eluted was calculated by dividing the target DNA band intensity in fraction S2 by the sum of the target DNA band intensities in fractions P and S2. The fraction of total input DNA in a given fraction was determined by dividing the target DNA band intensity for the fraction by the sum of target DNA band intensities for fractions S1, P, and S2 (fraction W was not included in this calculation because it did not contain any detectable target DNA). In Figure 5, the efficiency of USER digestion was assessed by dividing digested band intensity by full length band intensity to obtain the ratio of digested to full-length band intensity. The USER digestion was quantified in this way because SYBR gold does not necessarily stain different DNA species equally. Because EMSA bands had long trails at the edge of the lane that could overlap for multiple bands, TEC purity was calculated using the internal width of the lane that did not include the trails at the edges.

Intensity traces were generated in ImageJ 1.51s by drawing a box around each lane and using the ‘plot profile’ command. Identical box sizes were used for all direct comparisons. Because the gel in Figure 6*C* (lanes 4–6) was slightly curved and the +RNase sample was separated from -RNase samples to avoid cross-contamination, the intensity traces were manually aligned by the position of the well. The manually aligned traces in 6C match the replicate shown in Fig. S4*A*, which did not require alignment.

### Reproducibility of the methods

EMSAs of three independent TEC preparations that were purified using the final validated protocol (detailed above under Purification of TECs by selective photoelution from streptavidin beads) with DNA template 2 (Table S2) are shown in Figure 4*B* and Fig. S2; EMSAs of two additional TEC preparations that used less competitor DNA but performed identically to the final protocol are also shown in Figure 4*B* and Fig. S2. EMSAs of three independent TEC preparations that were purified using the final validated protocol with DNA template 6 (Table S2) are shown in Figure 6*C* and Fig. S4; a fourth TEC preparation that used more competitor DNA but performed identically to the final protocol is also shown in Figure 6*C*.

## Data availability

All data are contained in the article as plotted values or representative gels. Source TIFF files are available from the corresponding author (E. J. S.) upon request.

## Supporting information

This article contains supporting information.

## Conflict of interest

The author declares that he has no conflicts of interest with the contents of this article.
