# Supplementary material for: Preparation of E. coli RNA polymerase transcription elongation complexes by selective photoelution from magnetic beads
Source: J Biol Chem. 2021 May 21;297(1):100812. doi: 10.1016/j.jbc.2021.100812 (PMC8212663; doi:10.1016/j.jbc.2021.100812)
Supplement: Supplemental Figures S1–S6 and Table S1–S3 [file mmc1.docx]

**Preparation of *E. coli* RNA polymerase transcription elongation complexes by selective photoelution from magnetic beads**

**Eric J. Strobel**

**Materials Included:**

Figure S1. Verification of 5’ PC Biotin photocleavage using a custom 365 nm UV microcentrifuge tube irradiator.

Figure S2. Comparison of TEC purification conditions.

Figure S3. Secondary structures of linker and ZTP riboswitch sequences.

Figure S4. Additional visualization for experiments describing the purification of TECs containing a 194 nt RNA.

Figure S5. Quality control for internally modified DNA template preparations.

Figure S6. Additional visualization for experiments describing the initial development, optimization, and validation of the TEC purification procedure.

Table S1. Oligonucleotides used in this study.

Table S2. DNA templates prepared for this study.

Table S3. DNA template sequences.


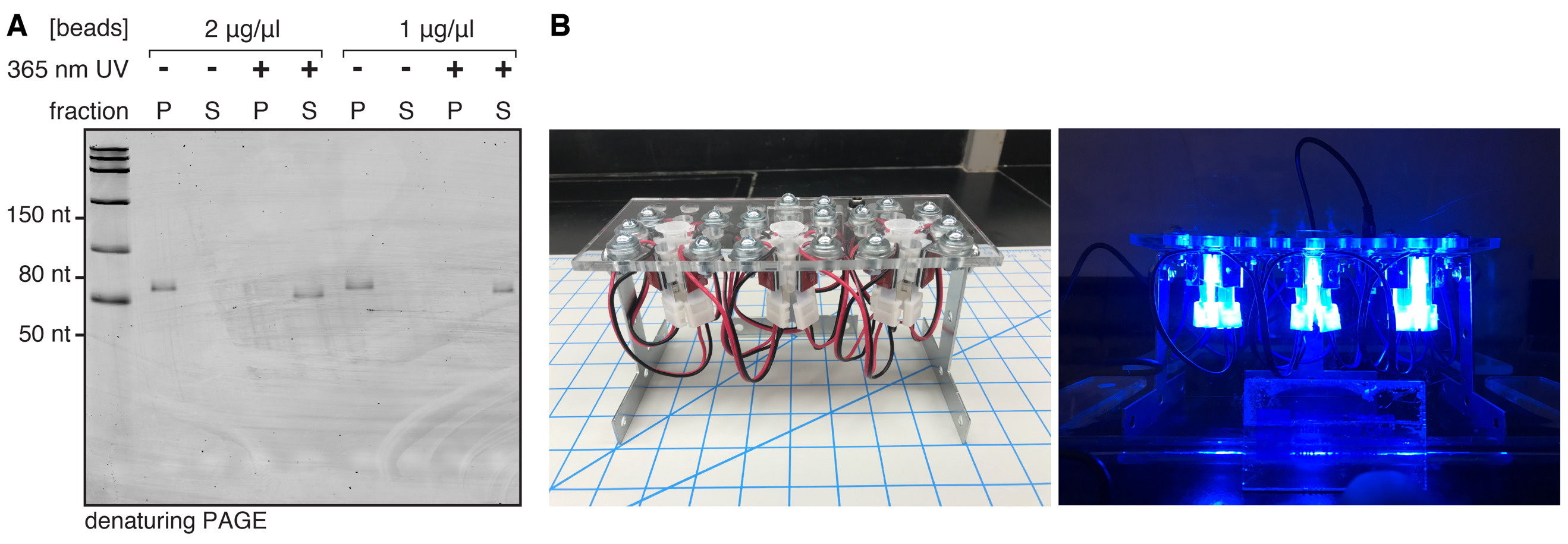


**Figure S1. Verification of 5’ PC Biotin photocleavage using a custom 365 nm UV microcentrifuge tube irradiator. (A)** Denaturing gel showing efficient 365 nm UV-dependent release of a 5’ PC biotin-modified oligonucleotide from two concentrations of streptavidin-coated magnetic beads. **(B)** Custom built 365 nm tube irradiator.


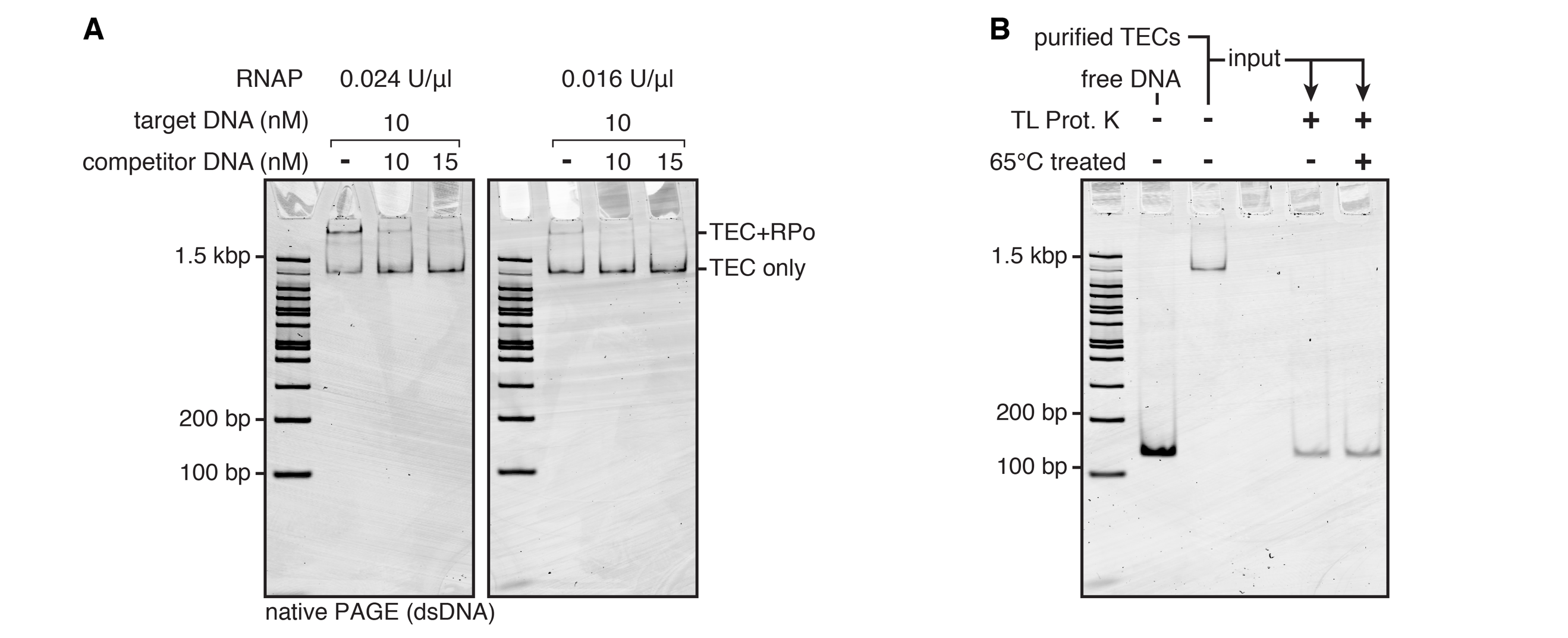


**Figure S2. Comparison of TEC purification conditions. (A)** EMSA of TECs purified using variable RNAP and competitor DNA concentrations. The gel containing 0.016 U/μl RNAP samples is a replicate of the corresponding gel shown in Figure 4B, shown here for comparison to the 0.024 U/μl RNAP samples and to illustrate the reproducibility of the purification. **(B)** Degradation of purified TECs using Thermolabile Proteinase K (TL Prot. K) followed by heat treatment at 65 °C.

**
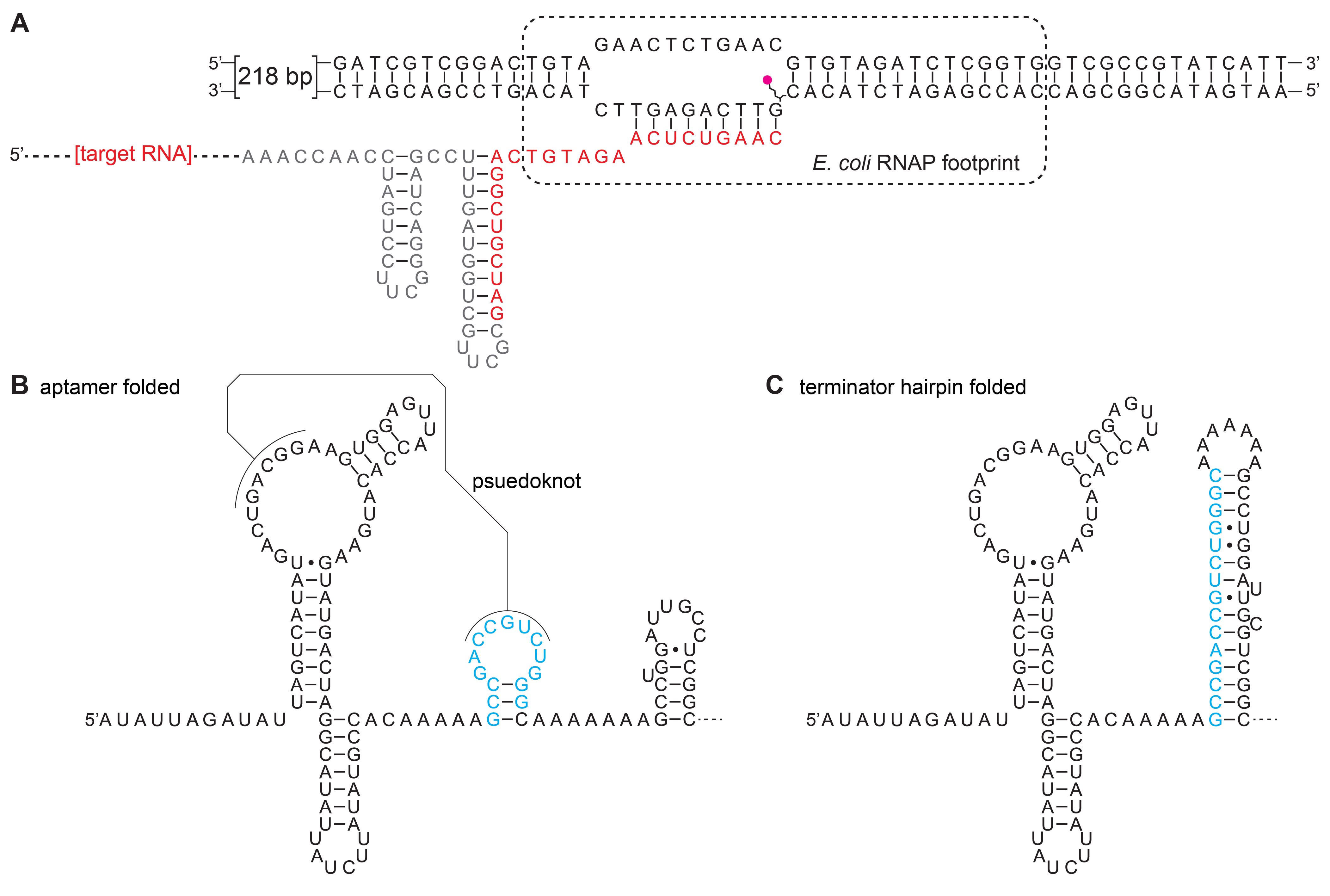
**

**Figure S3. Secondary structures of linker and ZTP riboswitch sequences.** Secondary structures of the (A) linker, **(B)** *pfl* ZTP riboswitch aptamer fold, and **(C)** *pfl* ZTP riboswitch terminator fold are shown.

**
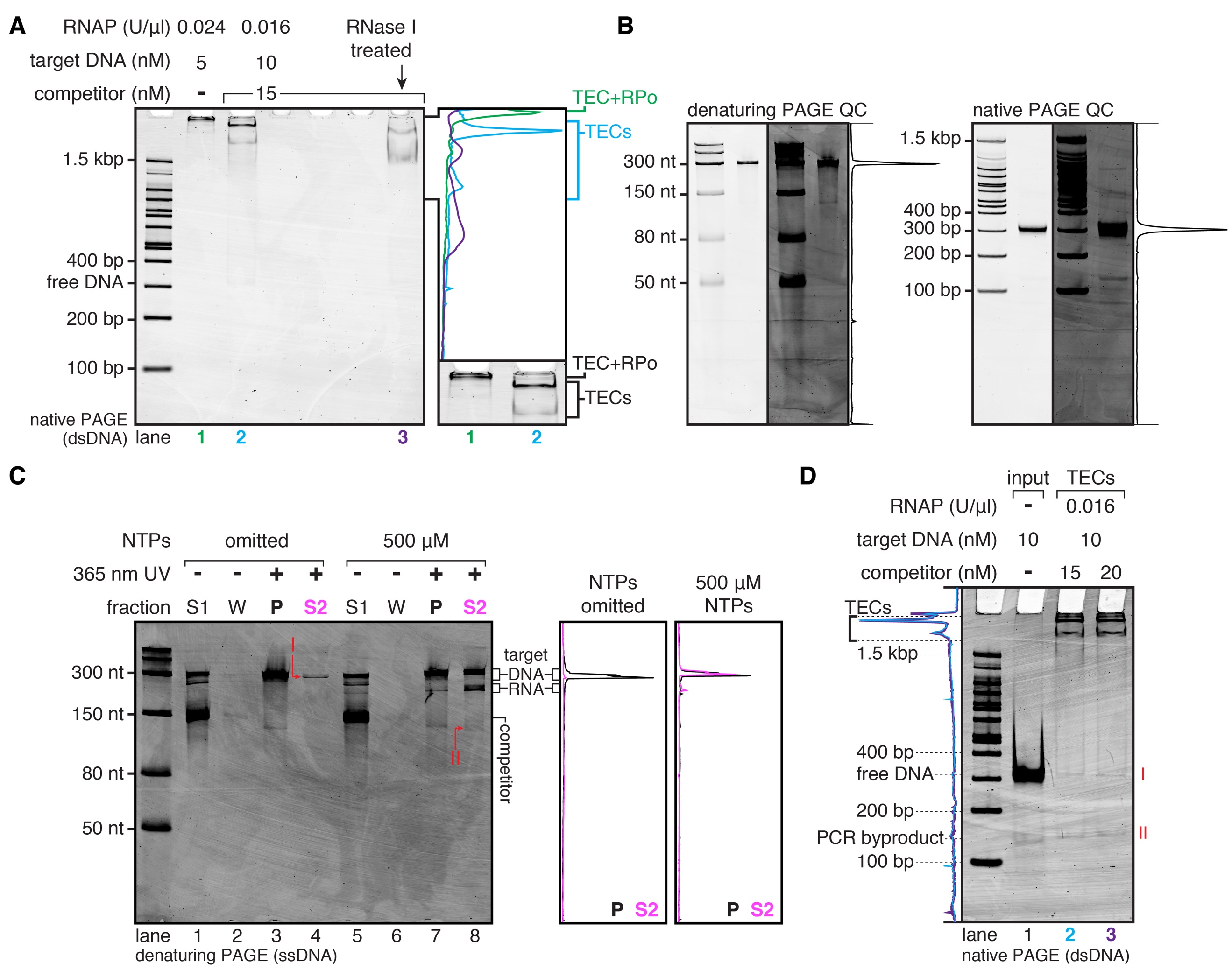
**

**Figure S4. Additional visualization for experiments describing the purification of** **TECs containing a 194 nt RNA.** The gels shown in panels C and D were also presented in Figure 6B, C and are shown here with an extreme grayscale setting to show low abundance bands. **(A)** Replicate of lanes 4, 5, and 6 in Figure 6C, showing EMSA analysis of purified TECs that contain a 194 nt RNA. Lane 1 contains TECs that were prepared using conditions that favor the formation of new open complexes after promoter escape. Lane 2 contains TECs purified using the optimized conditions. Lane 3 contains TECs that were purified using the optimized conditions then treated with RNase I (the corresponding sample in Figure 6C lane 6 was treated with a combination of RNase I, RNaseA, and RNase T1). **(B)** Denaturing and native PAGE quality control gels for the transcription template used in Figure 6. The gels are shown with two grayscale settings to reveal trace impurities in the preparation. Intensity traces are shown to the right of each gel to illustrate the abundance of the expected product relative to the impurities. **(C)** The gel from Figure 6B is shown with the grayscale adjusted to visualize low abundance bands. Band I is Target DNA that is eluted into Fraction S2 in the absence of NTPs, and most likely does not contain a functional internal biotin-TEG modification, either due to coupling efficiency or damage to the biotin moiety during oligonucleotide synthesis. Band II is a PCR byproduct that is present in trace amounts in the DNA template preparation shown in panel B. (**D**) The gel from Figure 6C (lanes 1-3) is shown with the grayscale adjusted to visualize low abundance bands. Bands I and II correspond to those described for panel C.

**
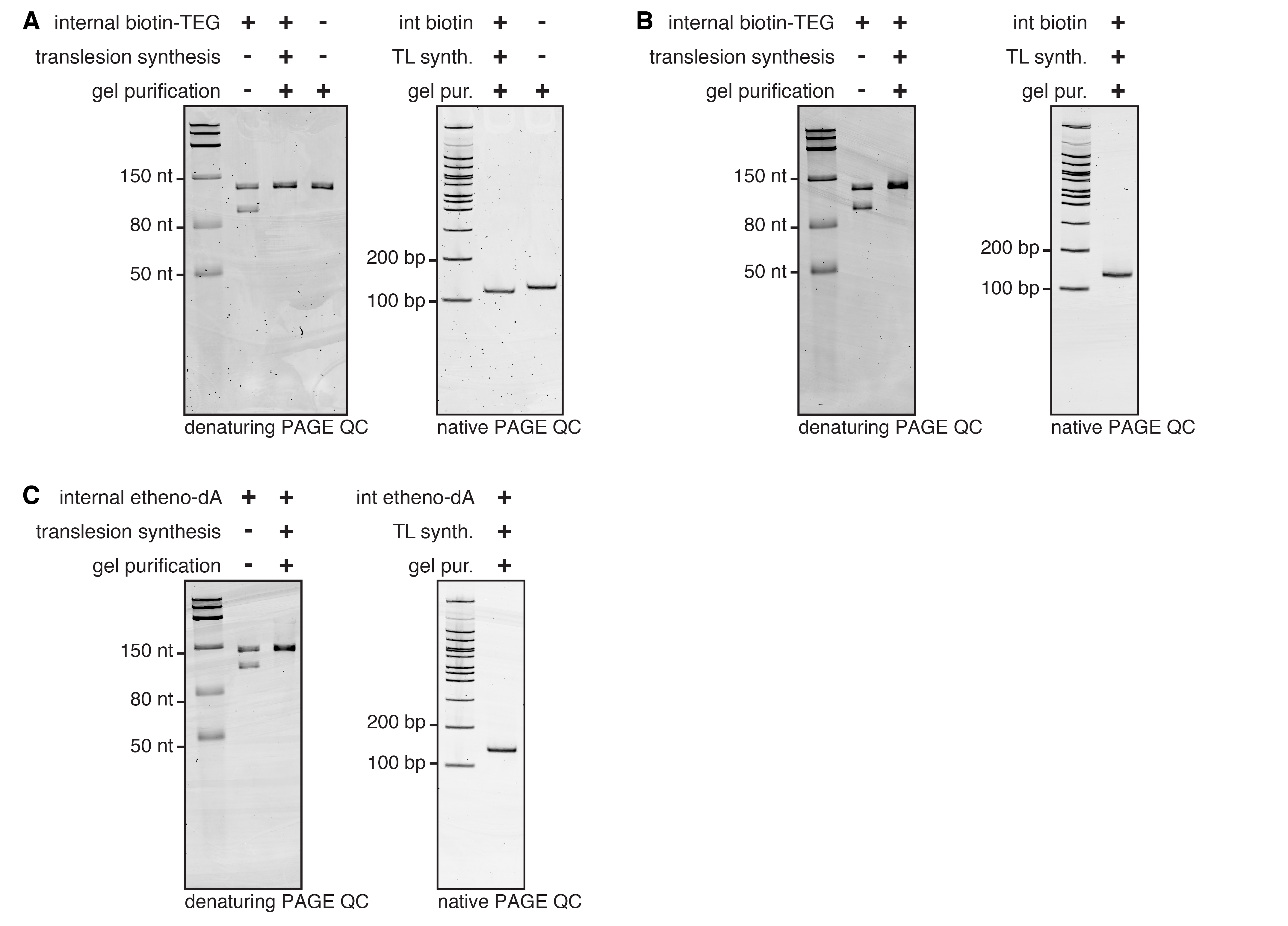
**

**Figure S5. Quality control for internally modified DNA template preparations.** Denaturing and non-denaturing quality control of **(A)** DNA template 1 , **(B)** DNA template 2, and **(C)** DNA template 3. Details of each DNA template preparation are available in Table S2. In Panel A, DNA Template 7 is used as a positive control DNA that did not contain an internal modification in both the denaturing and non-denaturing quality control gels. The presence of an internal modification causes a slight mobility shift relative to DNA without an internal modification.

**
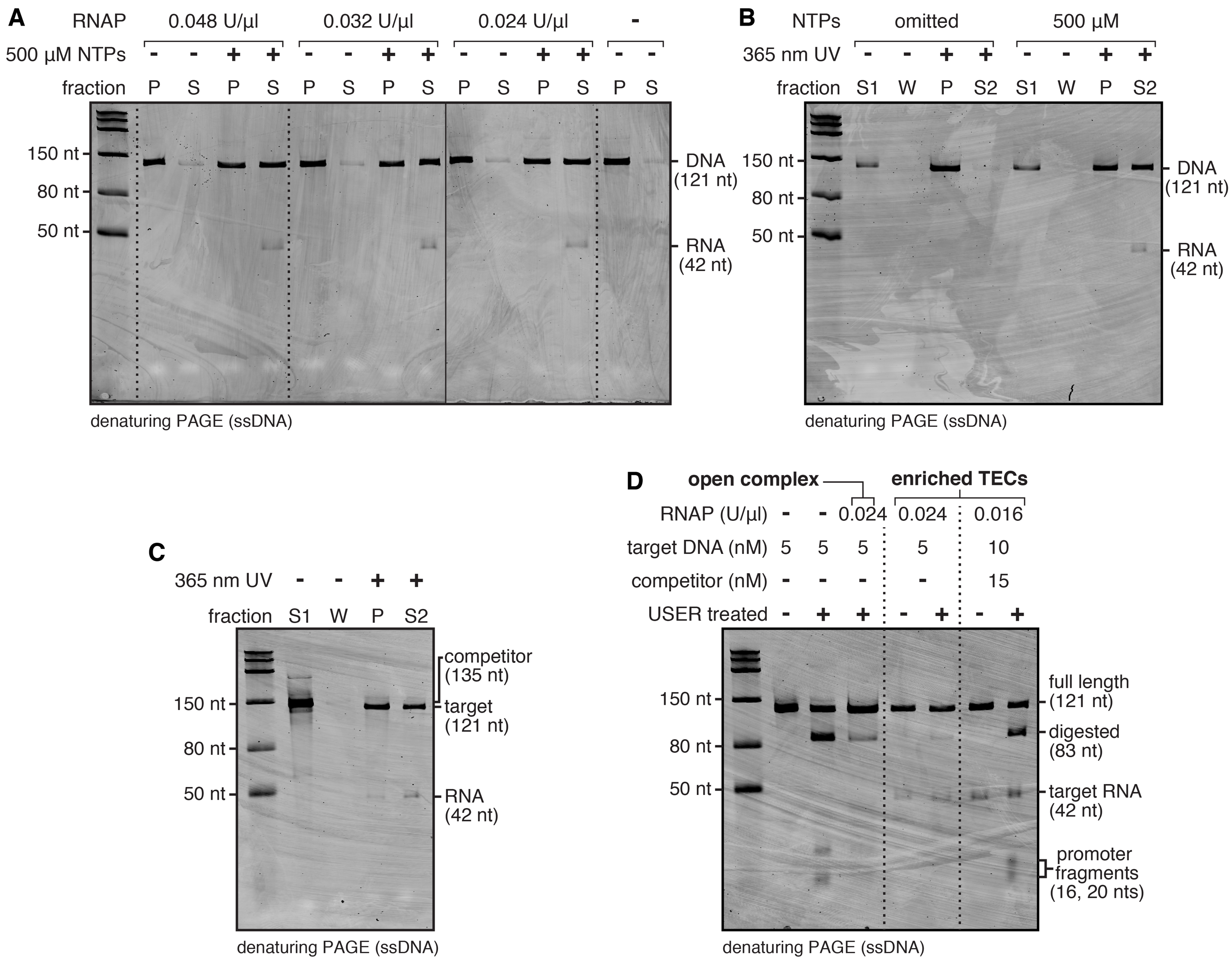
**

**Figure S6. Additional visualization for experiments describing the initial development, optimization, and validation of the TEC purification procedure.** Gels from main text figures **(A)** 2B, **(B)** 3B, **(C)** 4D, and **(D)** 5B in which the grayscale has been adjusted to better visualize the 42 nt Target RNA. Gels are presented with the same annotations as the main text versions.

**Table S1.** **Oligonucleotides used in this study.** Below is a table of oligonucleotides used for the preparation of *in vitro* transcription DNA templates. The modification codes defined below are used for compatibility with Integrated DNA Technologies ordering. DNA containing internal biotin-TEG and internal etheno-dA requires an off-catalog order.

/iBiotinTEG/: internal biotin-triethylene glycol

/iEth-dA/: internal etheno-dA

/ideoxyU/: internal deoxyuridine

/5PCBio/: 5’ photocleavable biotin

/5bioSG/: “standard” 5’ biotin

/5Cy5/: 5’ Cy5 dye

/5Cy3/: 5’ Cy3 dye

| ID | Name | Sequence | Purif. |
| --- | --- | --- | --- |
| TECD001 | dRP1iBio.R | AATGATACGGCGACCACCGAGATCTACAC/iBiotinTEG/GTTCAGAGTTCTACAGTCCGACGATC | HPLC |
| TECD002 | dRP1iEthdA.R | AATGATACGGCGACCACCGAGATCTACAC/iEth-dA/GTTCAGAGTTCTACAGTCCGACGATC | HPLC |
| TECD006 | PRA1_NoMod.F | TTATCAAAAAGAGTATTGACTCTTTTACCTCTGGCGGTGATAATGGTTGCAT | HPLC |
| TECD007 | PRA1_2dU.F | TTATCAAAAAGAGTATTGAC/ideoxyU/CTTTTACCTCTGGCGG/ideoxyU/GATAATGGTTGCAT | HPLC |
| TECD009 | PRA1_2dU_PCbio.F | /5PCBio//iSpC3/TTATCAAAAAGAGTATTGAC/ideoxyU/CTTTTACCTCTGGCGG/ideoxyU/GATAATGGTTGCAT | HPLC |
| TECD016 | wlk29.tmp | ACCTCTGGCGGTGATAATGGTTGCATGGAGAGGGATATAGGGAAAGTGGTGGACGATCGTCGGACTGTAGAACTCTGAAC | PAGE |
| LEGH67 | LZV3.tmp | ACCTCTGGCGGTGATAATGGTTGCATATTAGATATTAGTCGATCGTCGGACTGTAGAACTCTGAAC | PAGE |
| LEGI10 | dRP1_5bio.R | /5bioSG/AATGATACGGCGACCACCGAGATCTACACGTTCAGAGTTCTACAGTCCGACGATC | HPLC |
| EJS017 | dRP1_NoMod.R | AATGATACGGCGACCACCGAGATCTACACGTTCAGAGTTCTACAGTCCGACGATC | HPLC |
| EJS029 | dRP1_Cy5.R | /5Cy5/AATGATACGGCGACCACCGAGATCTACACGTTCAGAGTTCTACAGTCCGACGATC | HPLC |
| EJS030 | pRA1_2dU_Cy3.F | /5Cy3/TTATCAAAAAGAGTATTGAC/ideoxyU/CTTTTACCTCTGGCGG/ideoxyU/GATAATGGTTGCAT | HPLC |

**Table S2.** **DNA templates prepared for this study.** Below is a table of DNA templates that were prepared for this study, including the primers and template oligos used, DNA modifications, the PCR polymerase used, whether translesion synthesis was performed, which reaction clean up protocol was used (see Experimental Procedures), and the figures in which each DNA template was used.

| ID | Fwd Primer | Rev Primer | Template | Modifications | PCR  Polymerase | Translesion  Synthesis | Clean Up | Used in Fig(s) |
| --- | --- | --- | --- | --- | --- | --- | --- | --- |
| 1 | TECD007 | TECD001 | LEGH67 | -13,-30 dU;  Int biotin-TEG | Q5U | Yes | Gel Extracted | 2A, 2B, 4A, S5A, S6A |
| 2 | TECD009 | TECD001 | LEGH67 | 5’ PC biotin;  Int C3 spacer;  -13,-30 dU;  Int biotin-TEG | Q5U | Yes | Thermolabile ExoI  + PCR clean-up | 3B, 4B, 4D, 5B, S2A, S2B, S5B, S6B, S6C, S6D |
| 3 | TECD006 | TECD002 | TECD016 | Int etheno-dA | Q5 | Yes | Thermolabile ExoI  + PCR clean-up | 4B, 4D, 5B, 6B, 6C, S2A, S2B, S4A, S4C, S4D, S5C, S6C, S6D |
| 4 | EJS030 | EJS017 | LEGH67 | 5’ Cy3;  -13,-30 dU | Q5U | N/A | Thermolabile ExoI  + PCR clean-up | 4C |
| 5 | TECD007 | EJS029 | TECD016 | -13,-30 dU;  5’ Cy5 | Q5U | N/A | Thermolabile ExoI  + PCR clean-up | 4C |
| 6 | TECD009 | TECD001 | Gel-purified  linear DNA from pCES001 | 5’ PC biotin;  Int C3 spacer;  -13,-30 dU;  Int biotin-TEG | Q5U | Yes | Thermolabile ExoI  + PCR clean-up | 6B, 6C, S4A, S4B, S4C, S4D |
| 7 | TECD007 | LEGI10 | LEGH67 | -13,-30 dU;  5’ biotin | Q5U | N/A | Gel Extracted | S5A |

**Table S3.** **DNA template sequences.** Below is a table of containing the sequence of each DNA template. Fully annotated versions are available at Benching (See ‘Sequences’ in Experimental Procedures for hyperlinks).

| Name | Sequence of  DNA template(s) | Sequence |
| --- | --- | --- |
| LZV3  test | 1,2,4,7 | TTATCAAAAAGAGTATTGACTCTTTTACCTCTGGCGGTGATAATGGTTGCATATTAGATATTAGTCGATCGTCGGACTGTAGAACTCTGAACGTGTAGATCTCGGTGGTCGCCGTATCATT |
| wlk29  Competitor | 3, 5 | TTATCAAAAAGAGTATTGACTCTTTTACCTCTGGCGGTGATAATGGTTGCATGGAGAGGGATATAGGGAAAGTGGTGGACGATCGTCGGACTGTAGAACTCTGAACGTGTAGATCTCGGTGGTCGCCGTATCATT |
| pfl riboswitch, no poly-U, w/ linker | 6 | TTATCAAAAAGAGTATTGACTCTTTTACCTCTGGCGGTGATAATGGTTGCATATTAGATATTAGTCATATGACTGACGGAAGTGGAGTTACCACATGAAGTATGACTAGGCATATTATCTTATATGCCACAAAAAGCCGACCGTCTGGGCAAAAAAAGCCTGGATTGCGTCGGCAAACCAACCTAGTCCTTCGGGACTAGCCTTTGATGGTCGTTCGCGATCGTCGGACTGTAGAACTCTGAACGTGTAGATCTCGGTGGTCGCCGTATCATT |
